# The Heart of Fetal Growth Restriction: Congenital Heart Defects—An Analysis of Risk Factors and Morbidity Outcomes

**DOI:** 10.3390/life16091568

**Published:** 2026-09-19

**Authors:** Anca Adam-Raileanu, Delia Lidia Salaru, Ancuta Lupu, Elena Jechel, Mitica Ciorpac, Emil Anton, Laura Iulia Bozomitu, Stefana Maria Moisa, Oana Raluca Temneanu, Alice Grudnicki, Manuel Florin Rosu, Sorana Caterina Anton, Alin Horatiu Nedelcu, Magdalena Cuciureanu, Vasile Valeriu Lupu

**Affiliations:** Grigore T. Popa University of Medicine and Pharmacy, 700115 Iasi, Romania; anca.adam-raileanu@umfiasi.ro (A.A.-R.); delia.salaru@umfiasi.ro (D.L.S.); elena.jechel@yahoo.com (E.J.); mitica.ciorpac@umfiasi.ro (M.C.); laura.bozomitu@umfiasi.ro (L.I.B.); stefana-maria.moisa@umfiasi.ro (S.M.M.); temneanu.oana@umfiasi.ro (O.R.T.); alice.azoicai@umfiasi.ro (A.G.); florin.rosu@umfiasi.ro (M.F.R.); sorana.anton@umfiasi.ro (S.C.A.); alin.nedelcu@umfiasi.ro (A.H.N.); mag.cuciureanu@umfiasi.ro (M.C.); vasile.lupu@umfiasi.ro (V.V.L.)

**Keywords:** congenital heart defects, fetal growth restriction, cardiac phenotyping, comorbidity, burden

## Abstract

Background: Congenital heart disease (CHD) co-occurring with fetal growth restriction (FGR) is clinically important but incompletely characterized. We assessed the proportion and subtype spectrum of CHD in term-born children with FGR, and factors associated with its presence and complexity. Methods: Retrospective analysis of 375 term singleton children with documented FGR at a tertiary pediatric center. CHD required echocardiographic confirmation; isolated patent foramen ovale was excluded. Patients with multiple lesions were hierarchically classified as simple or complex. Multivariable logistic regression was restricted to characteristics available at or before birth. Results: CHD was confirmed in 96/375 children (25.6%): 71 (74.0%) simple, 25 (26.0%) complex. Atrial septal defect and patent ductus arteriosus were most frequent (each 45.8%), followed by patent foramen ovale (44.8%) and ventricular septal defect (32.3%). Severe FGR (aOR 2.012, 95% CI 1.222–3.313; *p* = 0.006) and genetic syndrome (aOR 5.969, 95% CI 2.565–13.889; *p* < 0.001) were independently associated with CHD presence. Within the CHD subgroup, genetic syndrome was also associated with complex disease (aOR 5.156, 95% CI 1.651–16.104; *p* = 0.005). Diagnosis occurred before six months in 95.8%. Hospitalization outcomes and comorbidity burden did not differ by complexity. Conclusions: FGR severity and genetic syndrome were both independently associated with the presence of a cardiac defect, and genetic syndrome was further associated with its complexity. These findings support targeted rather than universal echocardiographic assessment. Directionality cannot be established from postnatal data.

## 1. Introduction

Fetal growth restriction (FGR) is defined as the failure of a fetus to reach its genetically predetermined growth potential, typically defined as a birth weight below the 10th centile for gestational age with hemodynamic and Doppler evidence of placental insufficiency [1,2]. The clinically important distinction between FGR and constitutional small-for-gestational-age (SGA) lies in the substantially greater perinatal morbidity, long-term organ dysfunction, and cardiovascular programming that true FGR is associated with [1,3]. Internationally agreed staging systems, including the FIGO initiative on fetal growth, stratify FGR severity—birth weight below the 3rd centile designated as severe and between the 3rd and 10th centile as moderate—to guide clinical management and risk stratification [2]. Despite advances in antenatal surveillance, FGR continues to complicate 5–10% of pregnancies worldwide and remains a major contributor to perinatal mortality, preterm birth, and childhood morbidity [4,5,6].

The epidemiological burden of FGR extends beyond the neonatal period and may involve multiple organ systems, consistent with the Developmental Origins of Health and Disease (DOHaD) hypothesis [7,8]. In childhood and adolescence, FGR has been associated with altered growth trajectories and catch-up growth accompanied by distinct adiposity patterns and increased cardiometabolic risk [9,10], as well as neurodevelopmental impairment [5,11,12] and renal consequences, including reduced nephron endowment and increased susceptibility to hypertension and chronic kidney disease [3,13]. These multisystem consequences support the need for structured, multidisciplinary follow-up of children born with FGR.

The cardiovascular consequences of FGR represent among the most extensively studied manifestations of the DOHaD framework. Chronic placental insufficiency may produce hemodynamic redistribution—the ‘brain-sparing’ effect—which has been proposed to drive compensatory cardiac remodeling already evident in fetal life, characterized by increased ventricular afterload, altered myocardial strain, and subclinical dysfunction [14,15,16]. At the cellular level, chronic intrauterine hypoxia and oxidative stress have been hypothesized to trigger cardiomyocyte hypertrophy, impair sarcomere assembly, and alter calcium-handling proteins, potentially contributing to diastolic stiffness and reduced contractile reserve; elevated cortisol secondary to HPA-axis programming has also been proposed to further amplify myocardial fibrosis [17,18]. These changes may persist into childhood, adolescence, and adult life, with longitudinal echocardiographic studies reporting impaired left ventricular strain, diastolic dysfunction, and increased wall thickness proportional to FGR severity [19,20,21,22,23,24].

Beyond this acquired functional remodeling, congenital heart defects (CHDs)—the most common major birth defects, affecting 8–10 per 1000 live births globally and accounting for approximately 28% of all congenital anomalies [25,26,27]—also occur at elevated rates in the FGR population. The spectrum ranges from hemodynamically insignificant lesions to complex cyanotic malformations, with a multifactorial etiology encompassing chromosomal, genetic, maternal, and environmental determinants [28,29,30,31,32]; despite advances in prenatal screening, CHD continues to impose a lifelong management burden [13,33,34].

The intersection of FGR and structural CHD is of particular clinical importance, potentially reflecting a complex, bidirectional relationship rather than a single causal pathway. Epidemiological studies have demonstrated an inverse association between birth weight and CHD risk, with a recent systematic review confirming that low birth weight and SGA status are independently associated with an increased prevalence of structural cardiac defects across multiple subtypes [35]. The biological mechanisms potentially underlying this overlap may operate through intertwined, parallel pathways. First, shared etiologies may predispose to both conditions simultaneously: chromosomal and submicroscopic copy number variants—substantially more prevalent in FGR fetuses—have been linked to both pathologies [36,37], and genetic syndromes such as Trisomy 21 are independently associated with both FGR and specific defect subtypes, including complete atrioventricular canal defect [29]. Second, a dynamic interplay along the heart–placenta axis has been proposed. While severe hypoxic insults during early embryogenesis are generally incompatible with fetal viability, milder states of chronic suboptimal placental oxygenation and abnormal redox signaling are hypothesized to affect the developing heart. This localized oxidative stress may disrupt the expression of redox-sensitive transcription factors critical for cardiac morphogenesis (NKX2-5, GATA4, TBX5), potentially impairing septation, outflow tract rotation, and valvulogenesis [32,38]. Conversely, primary structural cardiac defects may alter fetal hemodynamics; these alterations—including reduced ventricular preload and altered shear stress—may independently modify structural remodeling of developing cardiac chambers and great vessels, whilst simultaneously compromising placental perfusion and potentially contributing to FGR [17,39]. These shared, bidirectional mechanisms may therefore contribute to the epidemiological overlap of these conditions through pathways that are potentially partially independent of the functional cardiac programming previously described.

The clinical burden of CHD in FGR children is compounded by the coexisting comorbidities of the growth-restricted phenotype—neurological impairment, renal anomalies, gastrointestinal dysfunction, and heightened infection susceptibility—increasing the complexity of perioperative and longitudinal management and driving early, repeated hospitalizations [12,13,34]. Despite this recognized interaction, the epidemiology of CHD within a FGR-defined pediatric cohort has not been comprehensively characterized: most existing studies either examine fetal cardiac function without addressing structural malformations, or investigate CHD in unselected birth cohorts without stratifying by FGR severity.

Our research group has previously characterized the metabolic, neurodevelopmental, and growth trajectories of a longitudinal FGR cohort from northeastern Romania [9,10], providing a well-defined platform for the investigation of cardiac outcomes in the same population. Building upon this foundation, the present study aims to investigate the epidemiology, risk factors, and clinical burden of congenital heart malformations in children with fetal growth restriction. The specific objectives were: (i) to describe the prevalence and spectrum of CHD subtypes; (ii) to identify independent factors associated with CHD presence; (iii) to characterize factors associated with structural complexity within the CHD subgroup; and (iv) to quantify clinical burden in terms of comorbidity accumulation and hospitalization outcomes—findings discussed in the context of optimizing prenatal counselling, postnatal echocardiographic screening, and multidisciplinary management of this high-risk cohort.

## 2. Materials and Methods

### 2.1. Study Design and Population

This study was conducted as a retrospective observational analysis. The research was based on previously recorded medical charts of patients aged 0–18 years, born at term from a singleton pregnancy, with a documented clinical history of FGR who underwent echocardiographic evaluation during continuous hospitalization at Saint Mary Children’s Hospital, Iași, Romania, between 1 January 2019 and 31 December 2023. Among these FGR patients, CHD status (present/absent) was determined by echocardiography, irrespective of the age at CHD diagnosis for those found to be affected. Isolated patent foramen ovale (PFO) cases were excluded from the CHD cohort, and all analyses were repeated accordingly. PFO was retained only when present in association with another structural cardiac defect.

For patent ductus arteriosus (PDA) and associated PFO, because of the retrospective nature of the epidemiological study based on available clinical records, we could not reliably determine the subsequent age at spontaneous closure of these lesions, nor could we ensure standardized longitudinal echocardiographic follow-up for all patients. Therefore, we did not attempt to estimate spontaneous closure rates or time-to-closure. The presence or absence of a documented lesion was interpreted according to the information available in the medical records at the time of assessment.

The study was conducted in accordance with the Declaration of Helsinki and approved by the local Ethics Committee (31602/26 October 2023; 383/27 January 2024).

**Figure 1:**
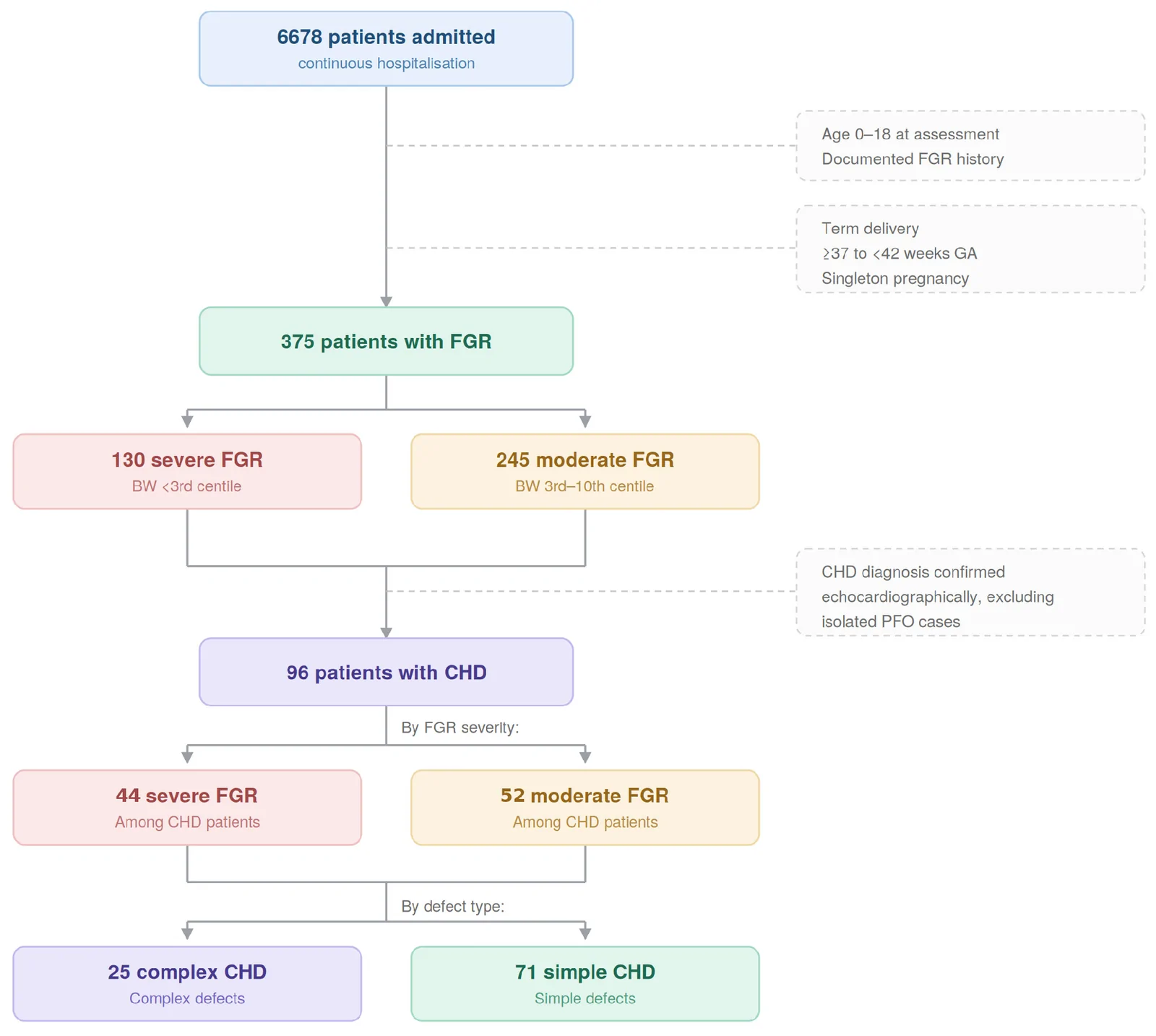
Flowchart of study group selection. FGR—fetal growth restriction; GA—gestational age; BW—birth weight; CHD—congenital cardiac heart defects; PFO—patent foramen ovale.

The primary analytic comparison was between children with a confirmed congenital heart malformation (CHD; *n* = 96) and those without CHD (*n* = 279), both drawn from the same FGR cohort. A nested subgroup analysis was conducted within the CHD group (*n* = 96), contrasting simple versus complex malformations. FGR was classified as severe if birth weight was below the 3rd centile, and moderate if birth weight fell between the 3rd and 10th centiles, in accordance with international consensus guidelines established by the International Federation of Gynecology and Obstetrics (FIGO) [2]. Centiles were derived using the INTERGROWTH-21st international newborn standards for weight by gestational age and sex [40], as recommended by the FIGO fetal growth restriction guideline. The rationale is described in Figure 1.

### 2.2. Data Collection and Variable Definitions

Data were extracted from electronic medical records into a structured database. Variables collected included: demographic characteristics (sex, residential milieu, maternal age at delivery, maternal education level); perinatal data (gestational age at birth, birth weight, FGR severity, mode of delivery); nutritional data (feeding type during the first six months of life); genetic background information (presence of genetic syndromes, Down syndrome (Trisomy 21) or other non-cardiac congenital malformations; comorbidities (neurologic, digestive, and renal); and hospitalization data (age at CHD diagnosis, early CHD diagnosis before six months, length of hospitalization [LOS]).

The cumulative comorbidity score was constructed as the unweighted sum of three binary (present/absent) comorbidity domains—neurologic, digestive, and renal—yielding a possible range of 0 to 3, with higher scores reflecting greater extra-cardiac comorbidity burden.

CHD was defined as the presence of any structural cardiac defect confirmed by echocardiography. Anatomical complexity of congenital heart disease (CHD) was stratified by adapting the established frameworks from the 2020 European Society of Cardiology (ESC) guidelines and the 32nd Bethesda Conference and ESC Guidelines on the Management of Adult Congenital Heart Disease (adapted for pediatric application) [41,42,43]. While these classification systems are traditionally applied to adult populations to guide long-term management, they were utilized in this pediatric cohort because the fundamental anatomical and structural complexity of a congenital lesion—established during embryogenesis—remains invariant across the lifespan. Utilizing these guidelines provides a robust, standardized, and reproducible anatomical hierarchy. To guarantee that all diagnostic categories were strictly mutually exclusive, patients presenting with multiple concurrent cardiac lesions were categorized hierarchically based exclusively on their most severe or structurally complex defect. Consequently, each patient was assigned to a single diagnostic tier, thereby eliminating category overlap or double-counting.

Following this hierarchical assignment rule, the cohort was dichotomized into simple and complex congenital heart disease categories. Simple defects included ventricular septal defect (VSD), atrial septal defect (ASD), patent ductus arteriosus (PDA), patent foramen ovale (PFO), aortic stenosis (AOS), pulmonary stenosis (PS), bicuspid aortic valve (BAV), coarctation of the aorta (COA), dextrocardia, and atrial septal aneurysm (ASA). Importantly, cases of isolated PFO were strictly excluded from the study cohort, as an isolated PFO is considered a normal physiological variant rather than a structural malformation; consequently, PFO was only included in the analysis when presenting concurrently with other structural defects. Complex defects included tetralogy or pentalogy of Fallot (TOF/POF), double outlet right ventricle (DORV), double-chambered right ventricle (DCRV), hypoplastic pulmonary artery (HPA), complete atrioventricular canal (CAVC), transposition of the great arteries (TGA), and common atrium (CA).

Maternal education was categorized into four ordinal levels: no studies or primary school (merged due to small cell size), gymnasium, high school, and university.

### 2.3. Statistical Analysis

All analyses were performed using Jamovi (version 2.7.33). Continuous variables are presented as mean ± standard deviation and categorical variables as absolute frequencies and percentages; normality was assessed using the Shapiro–Wilk test. Group comparisons (male vs. female; CHD present vs. absent) used Welch’s independent samples t-test for continuous variables and Pearson’s chi-square or Fisher’s exact test for categorical variables. Within the CHD subgroup (*n* = 96), simple vs. complex CHD comparisons used Mann–Whitney U, chi-square, or Fisher’s exact test with Bonferroni correction where appropriate. Statistical significance was set at *p* < 0.05. Four regression models were constructed: a binomial logistic regression predicting CHD presence in the full cohort (Model 1A; *n* = 375), a sensitivity analysis in the severe FGR subgroup, a binomial logistic regression predicting CHD complexity within the CHD subgroup (Model 1B; *n* = 96), and a linear regression predicting age at CHD diagnosis (Model 1C). Variables were selected based on bivariate *p* < 0.10 combined with a priori clinical relevance. Model 1C used log-transformed age due to skewed distribution.

A fifth model, a multivariable log-linear regression on length of hospitalization (log-transformed LOS as outcome; predictors: cardiac complexity, gestational age at birth, birth weight per 100 g increment, maternal education, genetic syndrome, and non-cardiac congenital malformation) was performed within the CHD subgroup (*n* = 96) to identify independent determinants of hospitalization duration. Residual normality, homoscedasticity, and influential observations (Cook’s distance) were assessed diagnostically.

Model fit for logistic regressions was assessed by likelihood ratio chi-square, Nagelkerke R^2^, and AUC-ROC; linear regression significance was assessed by F-test. Multicollinearity was evaluated using the variance inflation factor (VIF < 5). A Benjamini–Hochberg false discovery rate correction (q = 0.05) was applied across the full family of 102 subtype-level comparisons performed within the CHD subgroup (17 CHD subtypes × 6 outcome domains: genetic syndrome presence, Down syndrome presence, FGR severity, cumulative comorbidity score, age at CHD diagnosis, and length of hospitalization); primary regression-model outcomes were evaluated separately at α = 0.05 without adjustment. Three exploratory analyses were performed within the CHD subgroup: hierarchical clustering on CHD subtype co-occurrence data, correspondence analysis to visualize structural associations between subtypes and outcomes, and k-means latent class approximation to identify candidate subtype aggregates. These are hypothesis-generating and require confirmation in prospective cohorts.

To quantify optimism arising from evaluating model performance in the same sample used for model development, internal validation of Model 1A, its severe-FGR sensitivity analysis, and Model 1B was performed using bootstrap resampling (2000 resamples with replacement per model), following the method of Harrell et al. For each bootstrap sample, the logistic regression model was refit and its discriminative performance (AUC) and calibration (intercept and slope, from a logistic regression of observed outcome on the linear predictor) were evaluated both in the bootstrap sample (apparent performance) and in the original sample (test performance); the mean difference across resamples (“optimism”) was subtracted from the apparent, whole-sample estimate to yield an optimism-corrected estimate. The Brier score (0 = perfect calibration and discrimination) was corrected analogously. As a benchmark, the Brier score of a non-informative (null) model that always predicts the observed outcome prevalence equals *p*(1 − *p*), which for this cohort’s CHD prevalence (25.6%) is approximately 0.190. Bootstrap validation was performed in Python (statsmodels v0.14.6), as this procedure is not implemented in Jamovi.

## 3. Results

### 3.1. Cohort Characteristics and CHD Prevalence

The study cohort comprised 375 children with documented FGR, of whom 174 (46.4%) were males and 201 (53.6%) females. The cohort was predominantly rural (283/375, 75.5%). Mean gestational age at birth was 38.0 ± 0.29 weeks (near-uniform across the cohort), and mean birth weight was 2510 ± 186 g. Severe FGR (birth weight < 3rd centile) was present in 130 patients (34.7%) and moderate FGR in 245 (65.3%). Cesarean delivery was the predominant mode of birth (357/375, 95.2%). Baseline characteristics of the full cohort stratified by sex and CHD status are presented in Table 1.

Congenital heart malformation was identified in 96 of 375 patients (25.6%). The CHD-positive and CHD-negative groups were well-matched for sex (45.8% vs. 46.6% male; *p* = 0.897), residential milieu (*p* = 0.127), maternal age (*p* = 0.487), mode of delivery (*p* = 0.265), feeding type in the first six months (*p* = 0.310), and neurologic comorbidity (31.2% vs. 32.3%; *p* = 0.855). Significant differences between the CHD-positive and CHD-negative groups were observed for FGR severity (severe FGR, below the 3rd centile: 45.8% vs. 30.8%; *p* = 0.008), birth weight (2472 ± 179 vs. 2523 ± 187 g; *p* = 0.022), digestive comorbidity (15.6% vs. 30.8%; *p* = 0.004), renal comorbidity (13.5% vs. 35.1%; *p* < 0.001), and maternal education level (*p* = 0.043).

Within the CHD-positive subgroup (*n* = 96), age at CHD diagnosis differed significantly by sex, with male infants diagnosed earlier than female infants (1.27 ± 1.99 vs. 1.92 ± 2.53 months; *p* = 0.030). Length of hospitalization did not differ significantly by sex within this subgroup (5.31 ± 2.30 vs. 5.46 ± 3.49 days; *p* = 0.800).

### 3.2. CHD Subgroup: Spectrum of Cardiac Defects

**Figure 2:**
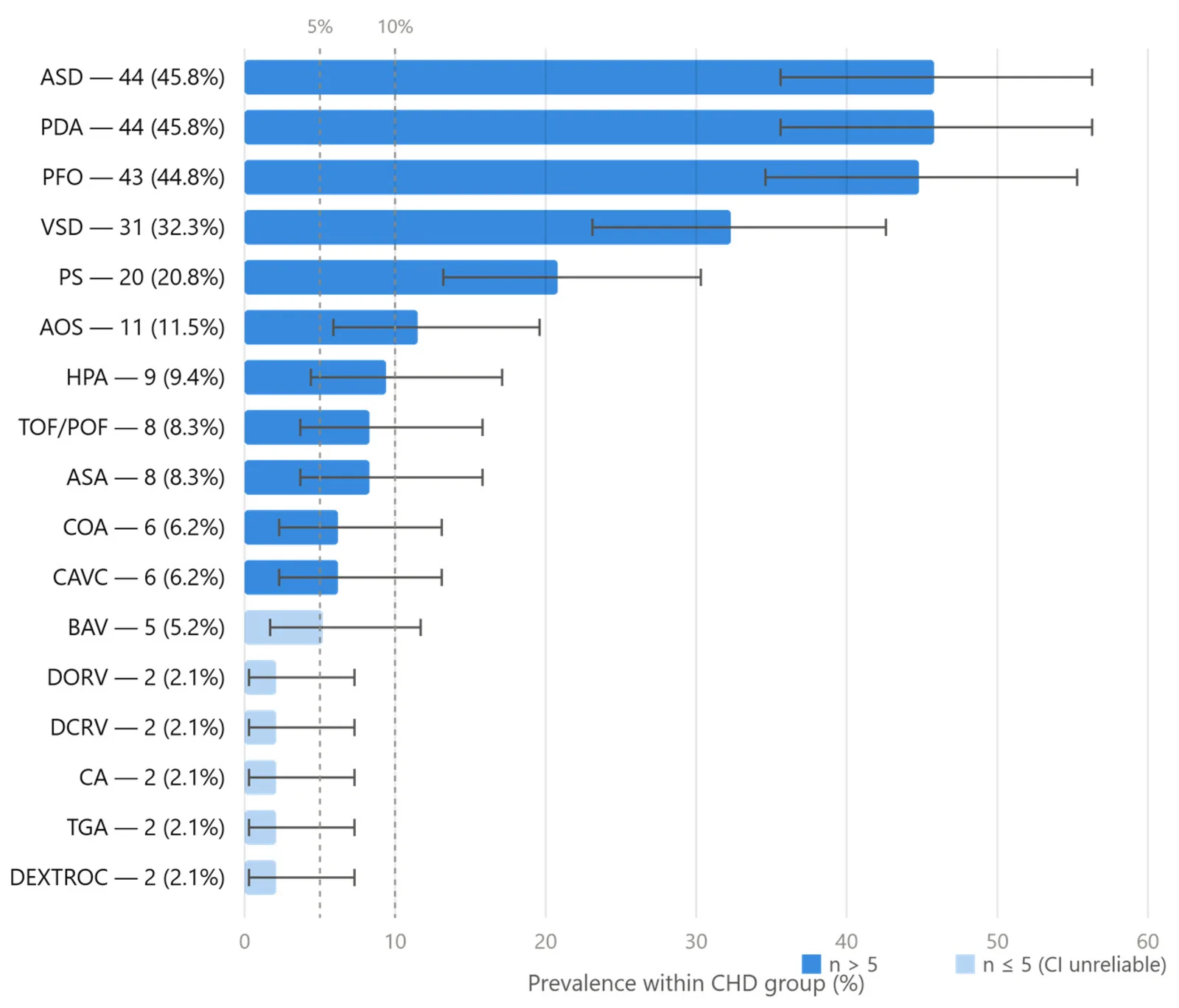
Prevalence of CHD subtypes within the CHD group with a coded defect (*n* = 96), sorted by descending frequency. Error bars = exact binomial 95% CIs. Dark blue = *n* > 5; light blue = *n* ≤ 5 (CIs unreliable). Vertical dashed lines indicate 5% and 10% prevalence thresholds. ASD—Atrial Septal Defect; PDA—Patent Ductus Arteriosus; PFO—Patent Foramen Ovale; VSD—Ventricular Septal Defect; PS—Pulmonary Stenosis; AOS—Aortic Stenosis; HPA—Hypoplastic Pulmonary Artery; TOF/POF—Tetralogy or Pentalogy of Fallot; ASA—Atrial Septal Aneurysm; COA—Coarctation of the Aorta; CAVC—Complete Atrioventricular Canal; BAV—Bicuspid Aortic Valve; DORV—Double Outlet Right Ventricle; DCRV—Double-Chambered Right Ventricle; CA—Common Atrium; TGA—Transposition of the Great Arteries; DEXTROC—Dextrocardia.

Shunting and septal lesions predominated: ASD and PDA were tied as the most frequent (both *n* = 44, 45.8%), closely followed by PFO (*n* = 43, 44.8%), and VSD (*n* = 31, 32.3%). Among more complex or obstructive lesions, pulmonary stenosis was present in 20 patients (20.8%) and aortic stenosis in 11 (11.5%). Complex defects including TOF/POF (n = 8, 8.3%), CAVC (*n* = 6, 6.2%), HPA (*n* = 9, 9.4%), and DORV, CA, TGA, and DEXTROC (each *n* = 2, 2.1%) accounted for a minority. Overall, 25 patients (26.0%) were classified as complex CHD and 71 (74.0%) as simple CHD. The prevalence of all subtypes with exact 95% binomial confidence intervals is shown in Figure 2.

**Figure 3:**
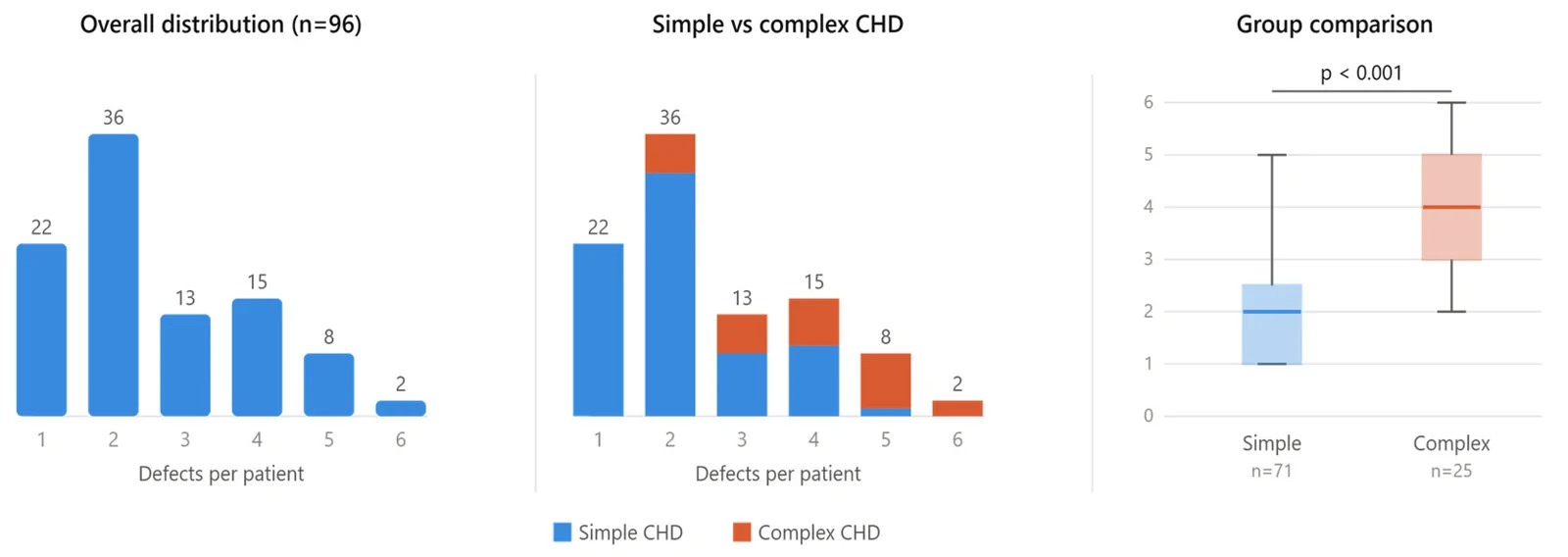
Number of concurrent CHD defects per patient. Left: overall distribution (*n* = 96; mean = 2.55). Center: distribution stratified by CHD complexity (blue = simple, coral = complex). Right: group comparison—complex CHD patients had significantly more concurrent defects than simple CHD patients. Both groups non-normal (Shapiro—Wilk, *p* < 0.05); Mann—Whitney U test used. *n* = 96.

The number of concurrent defects per patient ranged from 1 to 6, with a mean of 2.55. The distribution was right-skewed, with most patients carrying 1–2 defects (58/96, 60.4%). Complex CHD patients carried significantly more concurrent defects than simple CHD patients (median 4, IQR 3–5 vs. median 2, IQR 1–2.5; *p* < 0.001, Mann–Whitney U), as shown in Figure 3.

### 3.3. Empirical Subtype Association Patterns

**Figure 4:**
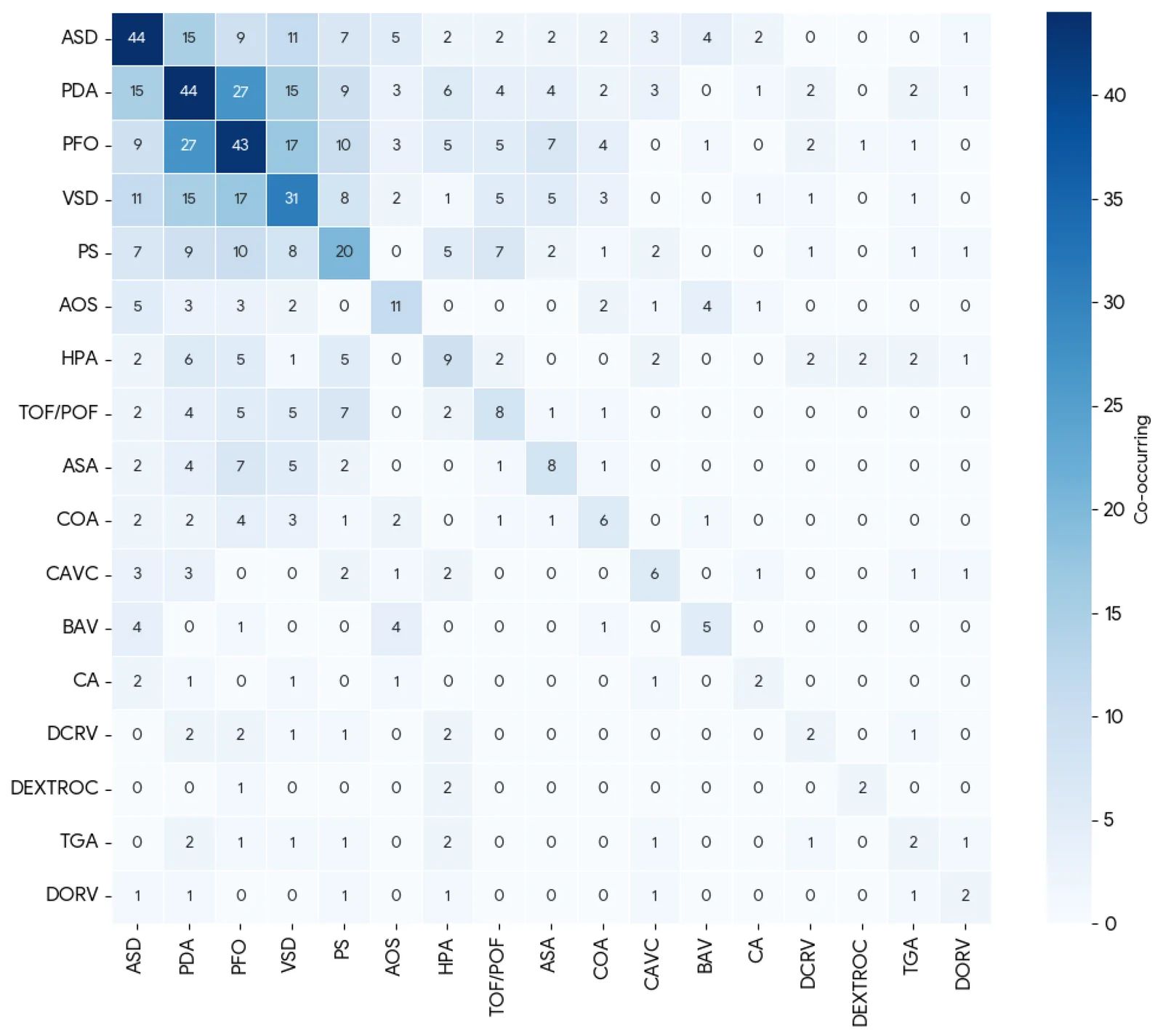
Co-occurrence matrix of CHD subtypes (*n* = 96). Each cell shows the number of patients sharing both defects simultaneously. Diagonal (grey) = total n for each subtype. Sorted by descending prevalence. Darker blue = greater co-occurrence. The PFO–PDA–ASD–VSD cluster is the most densely co-occurring group. ASD—Atrial Septal Defect; PDA—Patent Ductus Arteriosus; PFO—Patent Foramen Ovale; VSD—Ventricular Septal Defect; PS—Pulmonary Stenosis; AOS—Aortic Stenosis; HPA—Hypoplastic Pulmonary Artery; TOF/POF—Tetralogy or Pentalogy of Fallot; ASA—Atrial Septal Aneurysm; COA—Coarctation of the Aorta; CAVC—Complete Atrioventricular Canal; BAV—Bicuspid Aortic Valve; DORV—Double Outlet Right Ventricle; DCRV—Double-Chambered Right Ventricle; CA—Common Atrium; TGA—Transposition of the Great Arteries; DEXTROC—Dextrocardia.

Hierarchical clustering of 17 binary subtype variables using Jaccard distance and average linkage identified four empirical subtype aggregates (Table 2; Figure 4). Cluster 1 (AV Canal and Atrium defects: CAVC, CA) grouped defects arising from endocardial cushion developmental pathways. Cluster 2 (Outflow Tract and Complex: HPA, DORV, DCRV, TGA, DEXTROC) represented conotruncal malformations predominantly classified as complex CHD. Cluster 3 (Left-sided Obstructions: AOS, COA, BAV) captured left ventricular outflow tract lesions sharing hemodynamic physiology. Cluster 4 (Shunting Lesions: ASD, PDA, PFO, VSD, PS, TOF/POF, ASA) was the largest cluster comprising predominantly simple inter-chamber and vascular shunting defects. The co-occurrence heatmap (Figure 4) illustrates pairwise defect relationships, confirming that ASD–PDA–PFO–VSD form the most frequent core association. Given the relatively high intra-cluster Jaccard distances (0.81–0.86) and the modest silhouette score (0.207), these hierarchical aggregations should be interpreted strictly as descriptive, exploratory observations of subtype co-occurrence rather than validated biological aggregates. These patterns were further evaluated using correspondence analysis (see Section 3.11).

### 3.4. Independent Factors Associated with CHD in the Full FGR Cohort (Model 1A)

**Figure 5:**
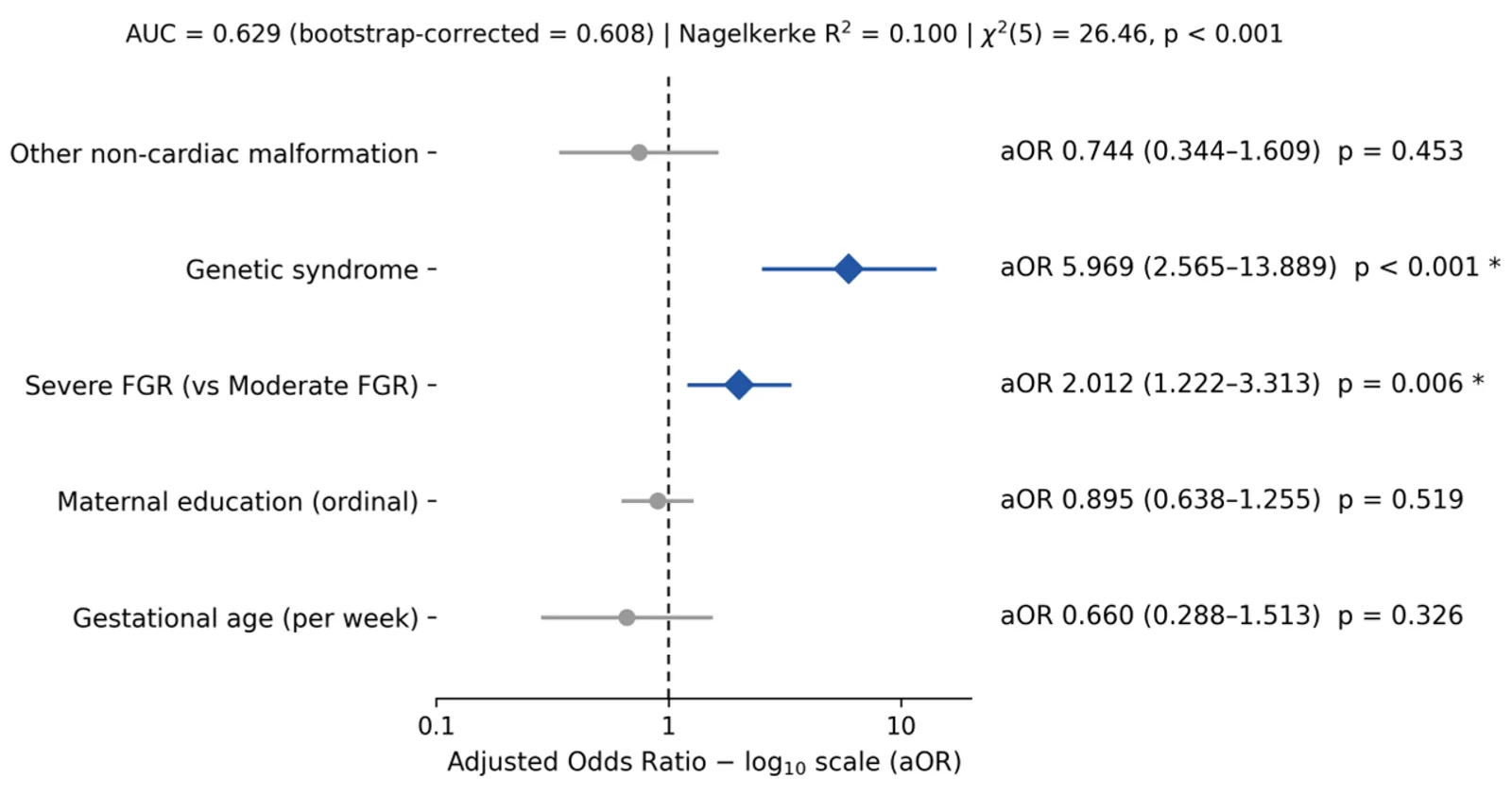
Forest plot of adjusted odds ratios (aOR) with 95% confidence intervals from multivariable logistic regression (Model 1A, *n* = 375). Blue diamonds = increased odds of CHD (* *p* < 0.05); grey = non-significant. Dashed vertical line = null effect (aOR = 1.0). *X*-axis on log_10_ scale.

Multivariable binomial logistic regression identified independent factors associated with the presence of CHD in the full cohort using exclusively pre-natal and at-birth baseline characteristics (Table 3, Figure 5). The model demonstrated appropriate overall fit (χ^2^(5) = 26.46, *p* < 0.001) and acceptable discrimination (AUC = 0.629, bootstrap-corrected = 0.608; Nagelkerke R^2^ = 0.100; AIC = 412.2; *N* = 375).

To minimize temporal leakage and avoid conditioning on variables occurring after birth or after CHD diagnosis, the primary multivariable logistic regression model was restricted strictly to characteristics available at or before birth. The baseline pre-natal model included gestational age at birth, maternal education, severity of fetal growth restriction, genetic syndrome, and non-cardiac congenital malformations (EPV = 19.2). Severe fetal growth restriction was independently associated with increased odds of CHD compared to moderate FGR (aOR = 2.01, 95% CI 1.22–3.31, *p* = 0.006), and presence of a genetic syndrome was independently and strongly associated with CHD (aOR = 5.97, 95% CI 2.57–13.89, *p* < 0.001). Gestational age at birth (aOR = 0.66, 95% CI 0.29–1.51, *p* = 0.326), maternal education level (aOR = 0.90, 95% CI 0.64–1.26, *p* = 0.519), and other non-cardiac congenital malformations (aOR = 0.74, 95% CI 0.34–1.61, *p* = 0.453) did not reach statistical significance. Internal validation using 2000 bootstrap resamples showed that the apparent AUC of 0.629 was modestly optimistic, yielding an optimism-corrected AUC of 0.608 (optimism = 0.021). The optimism-corrected Brier score was 0.181 (apparent = 0.175), and the calibration slope decreased from 1.00 (apparent, by construction) to 0.87 after correction (corrected calibration intercept = −0.13), indicating mild overfitting and a modest tendency toward overestimation of risk at the extremes, consistent with the model’s EPV of 19.2.

### 3.5. Sensitivity Analysis: Factors Associated with CHD Presence in Patients with Severe FGR (Model 1A Subgroup)

The primary pre-natal multivariable logistic regression model was repeated in the subgroup of patients with severe FGR (*n* = 130; CHD+ *n* = 44, CHD− *n* = 86) to assess whether pre-natal risk factor profiles differed by FGR severity (Table 4). The overall model did not reach statistical significance in this subgroup (χ^2^(4) = 7.38, *p* = 0.117; McFadden pseudo R^2^ = 0.045), reflecting the reduced power of the smaller sample. After Benjamini–Hochberg FDR correction across the four pre-natal predictors, genetic syndrome showed the strongest raw association with CHD (aOR = 5.30, 95% CI 1.28–22.05, *p* = 0.022) but did not survive FDR correction (*p*FDR = 0.087) and is therefore interpreted as exploratory rather than confirmatory. Gestational age at birth (aOR = 1.00, *p* = 0.998), maternal education (aOR = 0.71, *p* = 0.222), and other non-cardiac congenital malformation (aOR = 0.93, *p* = 0.882) did not reach significance. Internal validation using 2000 bootstrap resamples showed an apparent AUC of 0.602, falling to an optimism-corrected AUC of 0.552—close to the null value of 0.5, reflecting the limited discriminative value of this underpowered subgroup model. The optimism-corrected Brier score was 0.229 (apparent = 0.211), and the calibration slope fell substantially from 1.00 (apparent) to 0.65 after correction (corrected intercept = −0.24), indicating marked overfitting consistent with the subgroup’s reduced sample size and events-per-variable ratio (EPV = 11.0).

### 3.6. Genetic Syndromes, FGR Severity, and Comorbidities by CHD Subtype and Complexity

**Figure 6:**
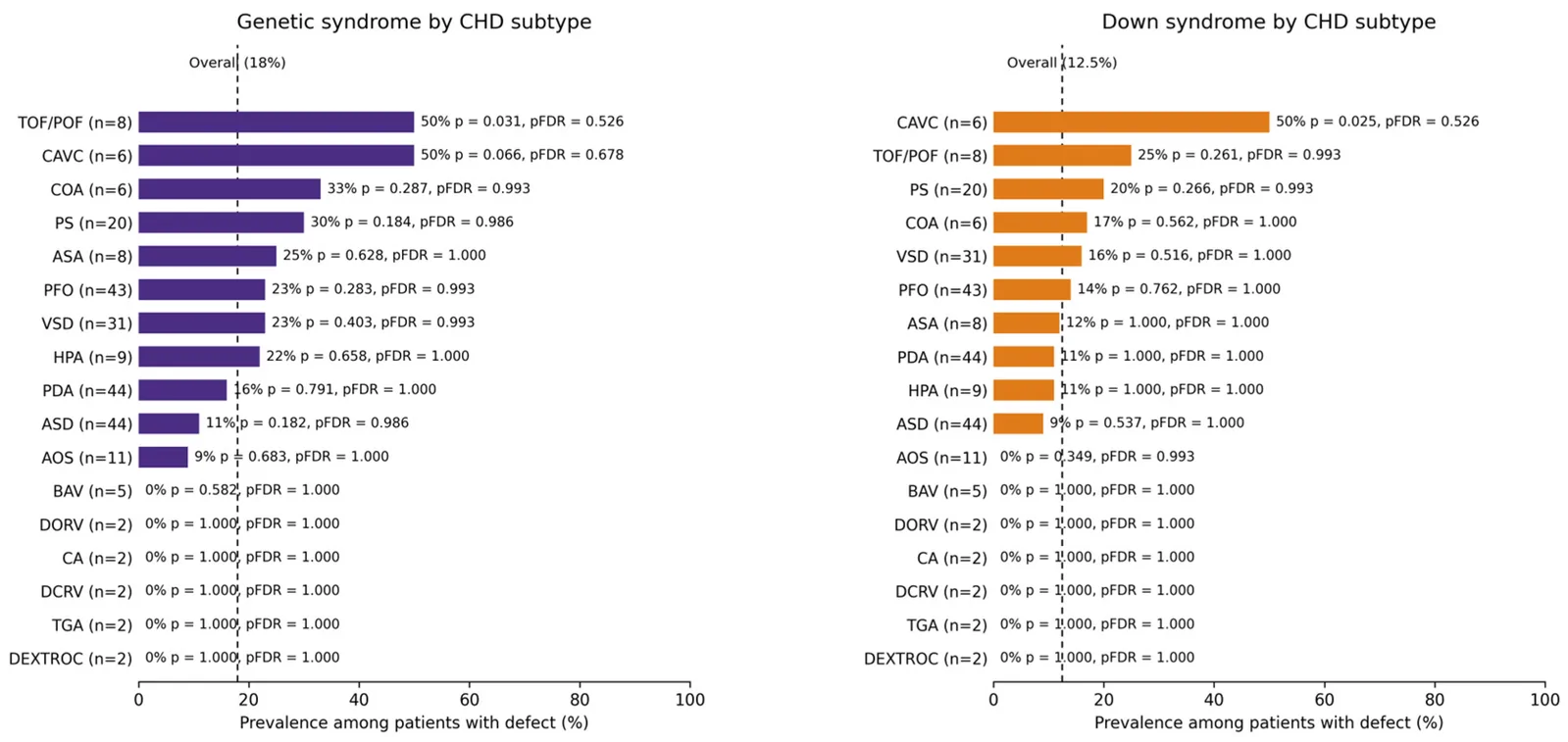
Prevalence of any genetic syndrome (Left Panel, purple) and Down syndrome (Right Panel, orange) among patients with each CHD subtype (*n* = 96). Sorted by ascending prevalence. Dashed line = overall CHD group prevalence (17.7% for genetic syndromes; 12.5% for Down syndrome). Raw *p*-values shown alongside Benjamini–Hochberg FDR-corrected *p*-values (*p*FDR), computed across the full family of 102 subtype-level comparisons (17 CHD subtypes × 6 outcome domains). Fisher’s exact test used throughout. CAVC (50.0%, *p* = 0.025, *p*FDR = 0.678) and TOF/POF (50.0%, *p* = 0.031, *p*FDR = 0.526) showed raw-significant but FDR-non-significant associations with genetic syndrome presence; CAVC was similarly associated with Down syndrome at the raw level (50.0%, *p* = 0.025, *p*FDR = 0.526). None survived correction across the full family of 102 subtype-level comparisons and are presented as exploratory. No asterisks are shown, as no subtype-level association in this panel reached FDR significance. *p* < 0.05. ASD—Atrial Septal Defect; PDA—Patent Ductus Arteriosus; PFO—Patent Foramen Ovale; VSD—Ventricular Septal Defect; PS—Pulmonary Stenosis; AOS—Aortic Stenosis; HPA—Hypoplastic Pulmonary Artery; TOF/POF—Tetralogy or Pentalogy of Fallot; ASA—Atrial Septal Aneurysm; COA—Coarctation of the Aorta; CAVC—Complete Atrioventricular Canal; BAV—Bicuspid Aortic Valve; DORV—Double Outlet Right Ventricle; DCRV—Double-Chambered Right Ventricle; CA—Common Atrium; TGA—Transposition of the Great Arteries; DEXTROC—Dextrocardia.

Within the updated cohort of 96 patients with CHD, 17 patients (17.7%) had a genetic syndrome, of whom 12 patients (12.5%) had Down syndrome (Trisomy 21). Down syndrome was most prevalent in patients with CAVC (3/6, 50.0%; *p* = 0.025, *p*FDR = 0.526). After Benjamini–Hochberg correction across the full family of 102 subtype-level comparisons, this association did not survive correction and is reported as exploratory. The overall prevalence of genetic syndromes was elevated in patients with TOF/POF (50.0%) and CAVC (50.0%), though these differences were not individually significant after correction for the small sample sizes involved (Figure 6).

**Figure 7:**
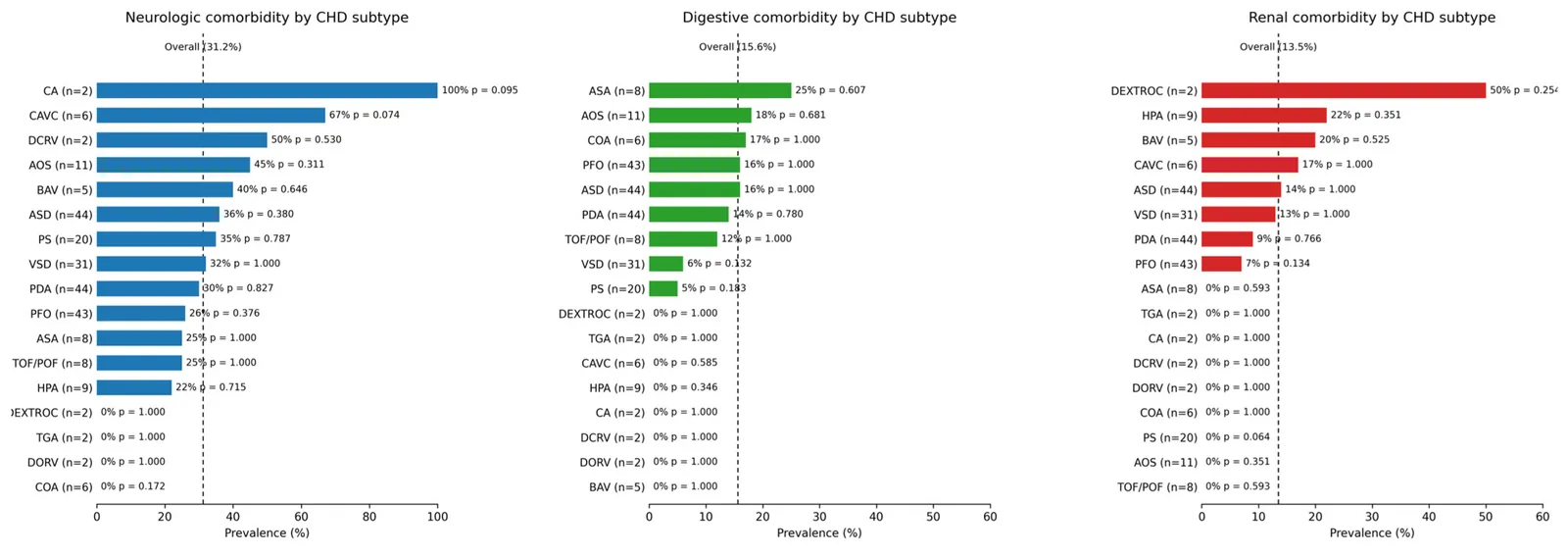
Prevalence of neurologic (blue), digestive (green), and renal (red) comorbidities by individual CHD subtype (*N* = 96). Sorted by descending prevalence within each panel. Dashed line = overall CHD group prevalence (31.2% for neurologic; 15.6% for digestive; 13.5% for renal). Fisher’s exact test used throughout. None of the individual subtype associations reached statistical significance after adjustment (*p* > 0.05). *p* < 0.05. ASD—Atrial Septal Defect; PDA—Patent Ductus Arteriosus; PFO—Patent Foramen Ovale; VSD—Ventricular Septal Defect; PS—Pulmonary Stenosis; AOS—Aortic Stenosis; HPA—Hypoplastic Pulmonary Artery; TOF/POF—Tetralogy or Pentalogy of Fallot; ASA—Atrial Septal Aneurysm; COA—Coarctation of the Aorta; CAVC—Complete Atrioventricular Canal; BAV—Bicuspid Aortic Valve; DORV—Double Outlet Right Ventricle; DCRV—Double-Chambered Right Ventricle; CA—Common Atrium; TGA—Transposition of the Great Arteries; DEXTROC—Dextrocardia.

Non-cardiac comorbidities were distributed similarly across CHD complexity groups after Bonferroni correction: neurologic comorbidity was present in 31.0% of simple vs. 32.0% of complex CHD patients (*p* = 1.000); digestive comorbidity in 18.3% vs. 8.0% (*p* = 1.000 Bonferroni); and renal comorbidity in 15.5% vs. 8.0% (*p* = 1.000 Bonferroni)—Figure 7.

Rates were consistently similar or slightly higher in the simple CHD group, indicating that extra-cardiac morbidity in this cohort is not driven by structural cardiac complexity. Unlike the initial uncorrected estimates, none of the individual subtype associations for renal comorbidity reached statistical significance after full evaluation (0% renal comorbidity in PS patients; *p* = 0.064), a finding that is now presented with appropriate caution (Table 5).

FGR severity did not differ significantly across most CHD subtypes. The notable exception was pulmonary stenosis, in which 17 of 20 patients (85.0%) had severe FGR, a significantly higher proportion than expected (*p* < 0.001, *p*FDR = 0.009)—the only subtype-level association surviving correction across the full family of 102 comparisons.

Cumulative comorbidity score (range 0–3) did not differ significantly between patients with any individual defect present versus absent. Coarctation of the aorta (COA) showed the lowest raw *p*-value (median 0.0 vs. 1.0; *p* = 0.058, *p*FDR = 0.678) but did not reach significance even before correction; no subtype showed a significant comorbidity-score difference.

### 3.7. Factors Associated with Complex vs. Simple CHD (Model 1B)

Multivariable logistic regression within the CHD subgroup (*N* = 96) was performed to evaluate factors associated with complex CHD (Table 6). The overall model reached statistical significance (*χ*^2^(2) = 8.34, *p* = 0.015; McFadden R^2^ = 0.076; AUC = 0.659). With 25 complex CHD events across two parameters, the events-per-variable ratio was adequate (EPV = 12.5).

In this multivariable specification, the presence of a genetic syndrome was significantly associated with complex CHD (aOR = 5.156, 95% CI 1.651–16.104, *p* = 0.005). However, as highlighted by methodological considerations regarding alternative specifications, this association should be interpreted cautiously within an exploratory framework rather than as definitive causal determination. Other non-cardiac congenital malformations were not independently associated with CHD complexity (aOR = 2.292, 95% CI 0.592–8.866, *p* = 0.230). Internal validation using 2000 bootstrap resamples confirmed that this apparent discrimination was only mildly optimistic (apparent AUC = 0.659; optimism-corrected AUC = 0.636; optimism = 0.022), with an optimism-corrected Brier score of 0.188 (apparent = 0.175) and a calibration slope of 0.93 after correction (apparent = 1.00), consistent with the model’s limited events-per-variable ratio (EPV = 12.5). While this indicates the model is not substantially overfit, its overall discrimination remains modest in absolute terms (AUC < 0.70); the bootstrap results therefore reinforce, rather than contradict, the exploratory interpretation above—the genetic syndrome–complex CHD association is statistically stable across resamples but should still not be read as evidence of strong predictive utility.

### 3.8. Hospitalization Outcomes in the CHD Subgroup

**Figure 8:**
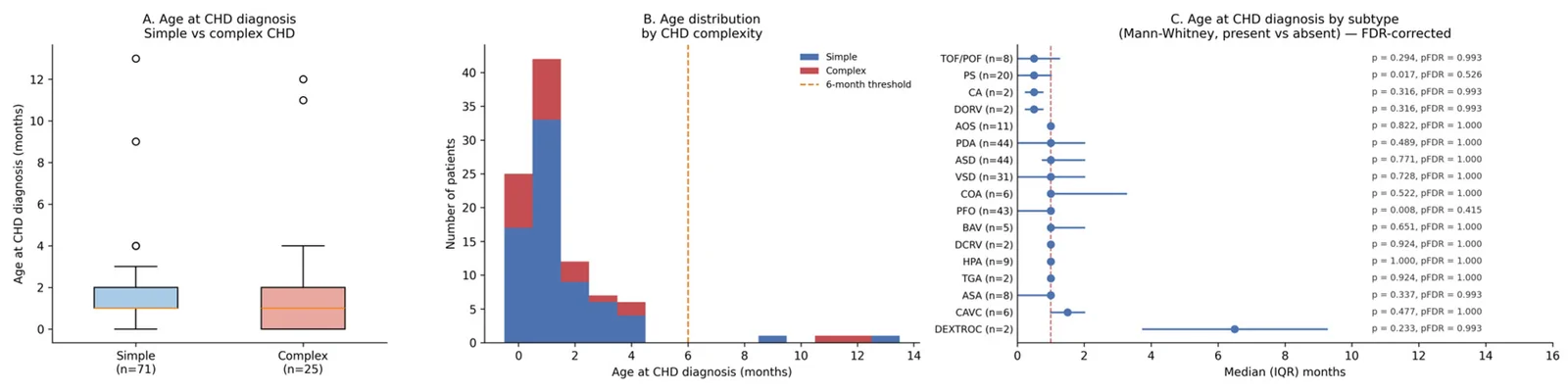
Age at CHD diagnosis in the CHD group (*n* = 96). Panel (**A**): Simple vs. complex CHD comparison (Mann–Whitney, *p* = 0.874, ns). Panel (**B**): Age distribution by CHD complexity with 6-month threshold (orange dashed line). Panel (**C**): Age at CHD diagnosis by individual CHD subtype (sorted by median; present vs. absent;). Panel (**C**) *p*-values are raw Mann–Whitney *p*-values with Benjamini–Hochberg FDR-corrected *p*-values (*p*FDR), computed across the full family of 102 subtype-level comparisons. PFO (*p* = 0.008, *p*FDR = 0.415) and PS (*p* = 0.017, *p*FDR = 0.526) showed raw-level associations with earlier diagnosis that did not survive FDR correction across the full family of 102 subtype-level comparisons and are reported as exploratory. Blue dots = median age at CHD diagnosis for each subtype; horizontal lines = interquartile range (IQR); ASD—Atrial Septal Defect; PDA—Patent Ductus Arteriosus; PFO—Patent Foramen Ovale; VSD—Ventricular Septal Defect; PS—Pulmonary Stenosis; AOS—Aortic Stenosis; HPA—Hypoplastic Pulmonary Artery; TOF/POF—Tetralogy or Pentalogy of Fallot; ASA—Atrial Septal Aneurysm; COA—Coarctation of the Aorta; CAVC—Complete Atrioventricular Canal; BAV—Bicuspid Aortic Valve; DORV—Double Outlet Right Ventricle; DCRV—Double-Chambered Right Ventricle; CA—Common Atrium; TGA—Transposition of the Great Arteries; DEXTROC—Dextrocardia.

Hospitalization characteristics are summarized in Table 7 and compared across complexity groups. The overall median age at CHD diagnosis in the CHD group was 1.0 month (IQR 0–2), with a mean of 1.63 months. Patients with complex CHD showed a slightly higher mean age at CHD diagnosis (1.96 months) compared to simple CHD (1.51 months), but this difference was not statistically significant (Mann–Whitney U = 906, *p* = 0.874). The rate of early CHD diagnosis (<6 months) was uniformly high in both groups: 97.2% in simple CHD versus 92.0% in complex CHD (*p* = 0.277)—Figure 8.

**Figure 9:**
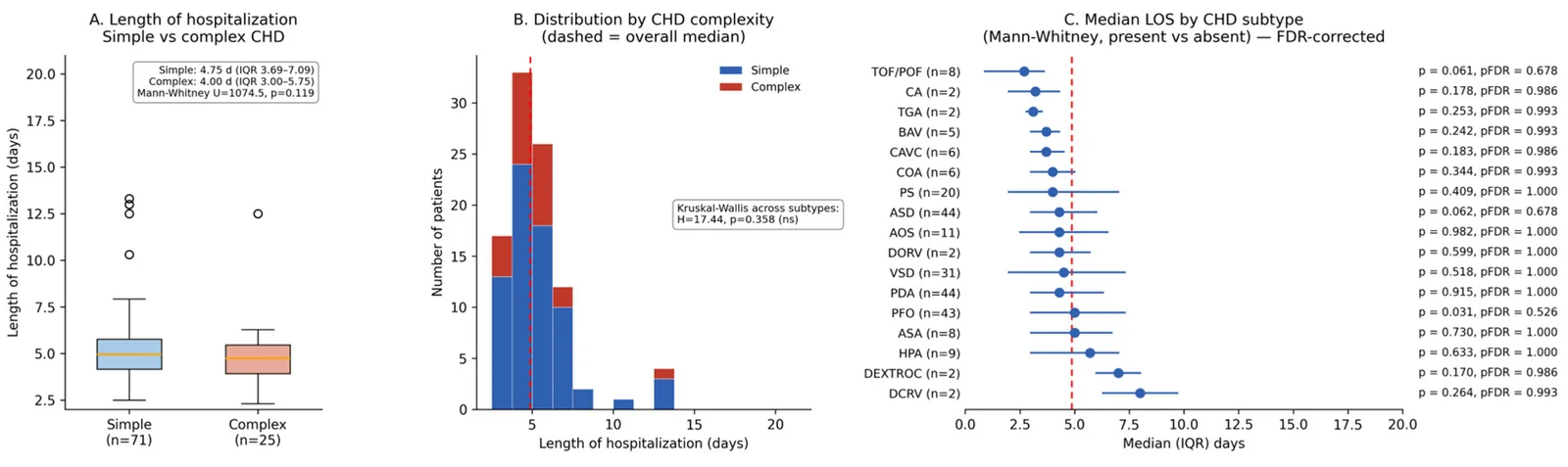
Length of hospitalization (LOS) in the CHD group (*n* = 96). Panel (**A**): Simple vs. complex CHD comparison—boxplot with jitter (Mann–Whitney, *p* = 0.119, ns). Circles = outlier data points on the boxplot—individual patients whose length of hospitalization falls beyond 1.5× the interquartile range (IQR) from the box edges—this is the standard boxplot convention. Panel (**B**): LOS distribution histogram by CHD complexity. The red dotted vertical line: the overall cohort median length of hospitalization. Panel (**C**): Median LOS by individual CHD subtype (present vs. absent). Panel (**C**) *p*-values are raw Mann–Whitney *p*-values with Benjamini–Hochberg FDR-corrected p-values (*p*FDR), computed across the full family of 102 subtype-level comparisons. PFO showed a raw-significant but FDR-non-significant association with longer LOS (*p* = 0.031, *p*FDR = 0.526). Kruskal–Wallis across all subtypes: H = 17.44, *p* = 0.358 (ns). No subtype survived FDR correction across the full family of 102 subtype-level comparisons; no asterisks are shown. Blue dots = median length of hospitalisation for each CHD subtype; horizontal lines = interquartile range (IQR); dashed red line = overall cohort median LOS. ASD—Atrial Septal Defect; PDA—Patent Ductus Arteriosus; PFO—Patent Foramen Ovale; VSD—Ventricular Septal Defect; PS—Pulmonary Stenosis; AOS—Aortic Stenosis; HPA—Hypoplastic Pulmonary Artery; TOF/POF—Tetralogy or Pentalogy of Fallot; ASA—Atrial Septal Aneurysm; COA—Coarctation of the Aorta; CAVC—Complete Atrioventricular Canal; BAV—Bicuspid Aortic Valve; DORV—Double Outlet Right Ventricle; DCRV—Double-Chambered Right Ventricle; CA—Common Atrium; TGA—Transposition of the Great Arteries; DEXTROC—Dextrocardia.

Length of hospitalization showed a non-significant trend toward shorter stays in complex CHD patients (median 4.00, IQR 3.00–5.75 days) compared to simple CHD (median 4.75, IQR 3.69–7.09 days; *p* = 0.119). At the subtype level, patients with PFO showed a numerically longer LOS than those without (mean 6.16 vs. 4.78 days; *p* = 0.031, *p*FDR = 0.526), and those with pulmonary stenosis showed a numerically earlier diagnosis (median 0.5 months vs. the group overall; *p* = 0.017, *p*FDR = 0.526). Neither association survived Benjamini–Hochberg correction across the full family of 102 subtype-level comparisons and both are reported as exploratory rather than significant. No other individual subtype showed a difference in LOS or diagnosis timing. The global Kruskal–Wallis test across all subtype groups for LOS was non-significant (H = 17.44, *p* = 0.358)— Table 7, Figure 9.

Multivariable Log-Linear Length of Stay (LOS) Model was used to identify independent determinants of hospitalization duration within the CHD cohort (*N* = 96), a multivariable log-linear regression model was performed. The model evaluated cardiac complexity, gestational age at birth, birth weight, maternal education, presence of genetic syndromes, and non-cardiac congenital malformations. Residual normality, homoscedasticity, and goodness-of-fit were verified via residual diagnostic plots. Cook’s distance statistics confirmed the absence of undue influential observations (max Cook’s D < 0.08). Cardiac complexity did not show a statistically significant independent association with hospitalization duration (exp(β) = 0.89, 95% CI 0.70–1.12, *p* = 0.302). Birth weight, expressed per 100 g increment, was independently associated with LOS (exp(β) = 1.06, 95% CI 1.003–1.120, *p* = 0.038): each additional 100 g of birth weight was associated with a 6% increase in geometric mean LOS. The overall model did not reach statistical significance (F(5,90) = 2.01, *p* = 0.084; R^2^ = 0.101), and this individual association should therefore be interpreted with caution pending replication in a larger sample.

### 3.9. Factors Associated with Age at CHD Diagnosis (Model 1C)

Linear regression on log-transformed age at CHD diagnosis within the CHD group (*n* = 96) showed an overall non-significant model (F(7,88) = 1.33, *p* = 0.243; R^2^ = 0.095; adjusted R^2^ = 0.023), and no individual variable survived Benjamini–Hochberg FDR correction (Table 8). All findings from this model are therefore exploratory. Female sex showed a borderline positive association with later CHD diagnosis on unadjusted testing (β = 0.361, unadjusted *p* = 0.026; back-transformed: +43.4% later diagnosis vs. males), but this did not survive FDR correction (*p*(FDR) > 0.05) and should be considered hypothesis-generating. CHD complexity, FGR severity, genetic syndrome, and all three comorbidity domains were not significantly associated with age at CHD diagnosis, even before correction. These results suggest that timing of first hospitalization in FGR children with CHD is not strongly predicted by the clinical variables examined in this study.

Gestational age (zero variance: all patients born at 38 weeks) and mode of delivery (sparse cell: only *n* = 8 vaginal deliveries) were excluded from Model 1C due to mathematical constraints.

**Figure 10:**
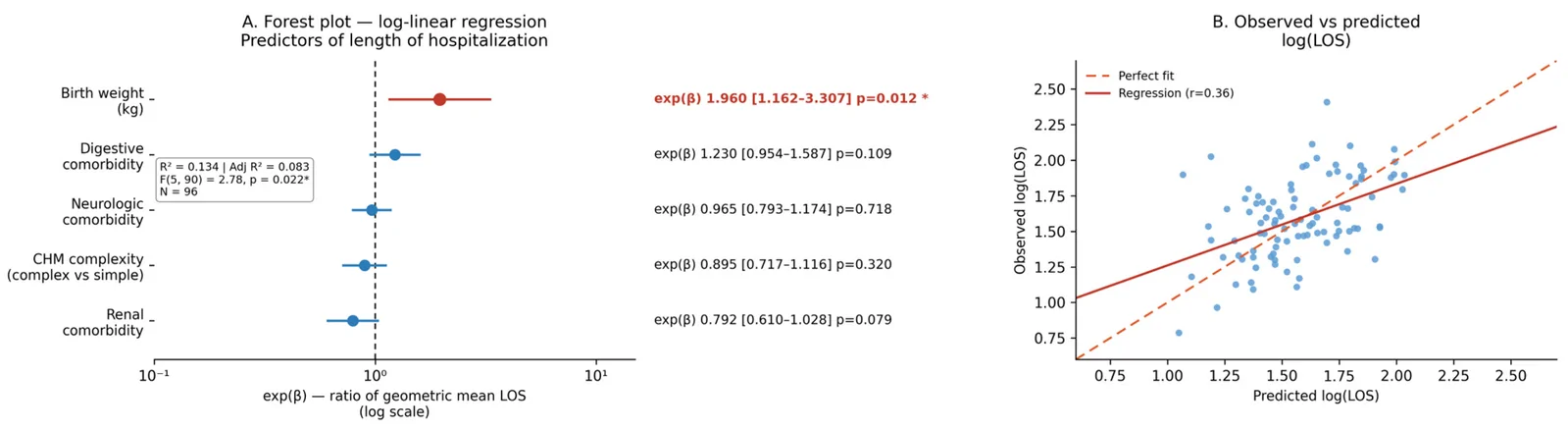
Log-linear regression predicting length of hospitalization (*n* = 96). Panel (**A**): Forest plot of exp(β) coefficients with 95% CIs. Birth weight (per 100 g increment) was the only significant factor (exp(β) = 1.06, 95% CI 1.003–1.120, *p* = 0.038 *): each additional 100 g of birth weight was associated with a 6% longer geometric mean LOS. All other factors were non-significant. Panel (**B**): Observed vs. predicted log(LOS) scatterplot (r = 0.35). Overall model: F(5,90) = 2.01, *p* = 0.084. Log-linear regression on OLS with log-transformed LOS. exp(β) = ratio of geometric mean LOS. GA excluded (zero variance). * *p* < 0.05. LOS: length of hospitalization; CHM: cardiac heart malformation.

Log-linear regression within the CHD subgroup (*n* = 96) identified birth weight, expressed per 100 g increment, as the only statistically significant individual factor associated with length of hospitalization (exp(β) = 1.06, 95% CI 1.003–1.120, *p* = 0.038), although the overall model did not reach global significance (*p* = 0.084). The observed vs. predicted scatter plot (Figure 10) confirms a modest predictive signal, with considerable individual-level variability around the regression line, consistent with the multifactorial and partially unmeasured determinants of hospitalization duration in FGR children with CHD.

### 3.10. Social, Nutritional, and Environmental Factors Within the CHD Subgroup

Feeding mode in the first six months of life did not differ significantly between simple and complex CHD groups (χ^2^(2) = 2.59, *p* = 0.274; Cramér’s V = 0.164). Notably, breastfeeding rates were somewhat higher in complex CHD patients (52.0%, *n* = 13/25) than in simple CHD patients (33.8%, *n* = 24/71), contrary to the hypothesis that cardiac complexity would reduce breastfeeding capacity, though the small complex CHD sample (*n* = 25) limits interpretation.

Residential milieu (rural vs. urban) showed no significant association with any CHD outcome: CHD complexity (*p* = 0.779), early CHD diagnosis rate (*p* = 1.000), age at CHD diagnosis (*p* = 0.175), LOS (*p* = 0.204), or feeding mode distribution (*p* = 0.354). CHD subtype prevalence did not differ by milieu for any of the 17 subtypes assessed. Maternal education was similarly unrelated to CHD complexity (χ^2^(3) = 0.67, *p* = 0.880) or to timing of CHD diagnosis (χ^2^(3) = 1.63, *p* = 0.653), though statistical power for the early CHD diagnosis analysis was very limited (only *n* = 5 patients had late CHD diagnosis).

### 3.11. Structural Associations and Subtype Aggregate Patterns—Exploratory Analyses

Correspondence analysis on the CHD subtype-by-comorbidity contingency table revealed two interpretable dimensions, 45.5% and 21.5%, respectively, accounting for 67.0% of total inertia. Dimension 1 (45.5% of inertia) separated complex and rare lesions (DORV, TGA, COA, HPA) at the positive pole from common shunting defects (ASD, PDA, PFO) at the negative pole, aligning with the simple/complex CHD classification axis. Dimension 2 (21.5% of inertia) separated renal and LVOT comorbidities (positive) from genetic syndrome and Down syndrome associations (negative). These patterns suggest that conotruncal and obstructive defects carry distinct comorbidity profiles from high-prevalence shunting lesions, even when formal statistical testing does not reach significance due to sample size constraints—Table 9.

K-means latent class approximation (k = 3; silhouette = 0.207) identified three candidate clinical aggregates: Aggregate 1 (PFO-dominant; *n* = 35, 36.5%), characterized by high genetic syndrome prevalence (31.4%) and the longest mean LOS (5.85 days); Aggregate 2 (ASD + PDA; *n* = 31, 32.3%), with the lowest genetic syndrome burden (12.9%) and intermediate LOS (4.73 days); and Aggregate 3 (Multi-shunt; *n* = 30, 31.3%), defined by higher numbers of concurrent defects (mean 3.24) and a lower neurologic comorbidity rate (24.2%). All three aggregates showed similar rates of complex CHD (~26%), similar birth weights, and a universal early CHD diagnosis pattern. Silhouette score = 0.207, indicating weak cluster separation; therefore, the aggregates should be considered hypothesis-generating rather than validated clinical classes; these aggregates should be considered hypothesis-generating for future confirmatory probabilistic latent class analysis—Table 10.

## 4. Discussion

### 4.1. Congenital Heart Disease in a Hospital-Ascertained FGR Cohort

The present study identified echocardiographically confirmed congenital heart disease in 96 of 375 children with fetal growth restriction (25.6%). This figure describes the proportion of CHD among FGR children admitted to a tertiary pediatric hospital and in whom cardiac assessment was performed when CHD was clinically or antenatally suspected. It is not a birth prevalence and cannot be converted into an excess-risk estimate, since the cohort was selected through tertiary-center ascertainment and lacks a population-based denominator of all FGR births. For the same reason, and because case definitions differ across studies, we refrain from numerical comparison with general-population birth prevalence figures [25] or with registry-based estimates in unselected pediatric populations [13,27]; the present estimate was obtained after exclusion of isolated patent foramen ovale and therefore reflects structural defects rather than physiological interatrial findings.

The association between impaired fetal growth and structural cardiac malformation is well documented, although estimates vary widely by study design, population and classification criteria. Wallenstein et al. [44] reported in a retrospective cohort that fetuses with CHD had significantly lower birth weights and higher rates of SGA classification than controls. Malik et al. [45], in a large case–control study from the National Birth Defects Prevention Study, found SGA to be associated with multiple CHD subtypes, particularly septal defects and conotruncal malformations. The systematic review and meta-analysis by Aliasi et al. [35] confirmed an inverse association between birth weight and isolated CHD across a pooled sample of over 100,000 subjects.

The direction of this relationship cannot be established in a postnatal, birth-weight-stratified cohort such as ours. Ghanchi et al. [46] set out three non-exclusive models that remain compatible with the available evidence: CHD altering fetal hemodynamics and oxygen delivery and thereby causing growth restriction; a shared genetic, placental, maternal or environmental determinant producing both aggregates; and early placental dysfunction during cardiogenesis contributing to the cardiac defect. Their meta-analysis reported SGA in approximately 20% of newborns with CHD and 14% of those with isolated CHD, against an expected 10%. Recent work on placental–cardiac cross-talk lends plausibility to the third model [47], while the classical literature on perturbation of cardiogenic gene networks during weeks three to eight of gestation [32,48,49] is equally compatible with a shared-mechanism interpretation; suboptimal placental oxygenation and oxidative stress, rather than severe hypoxia incompatible with embryonic viability, would be the relevant exposure under that model. We therefore describe the co-occurrence observed in this cohort as a bidirectional and multifactorial association, and we make no claim that growth restriction preceded or caused the cardiac lesion in these patients. No first-trimester biometry, crown–rump length or systematic fetal echocardiography was available, so the temporal sequence between placental insufficiency and structural lesion development remains unresolved by design.

### 4.2. Spectrum of Cardiac Defects and Burden of Concurrent Lesions

The defect spectrum was dominated by shunting and septal lesions: atrial septal defect and patent ductus arteriosus were equally frequent (each 45.8%), followed by patent foramen ovale in association with another structural lesion (44.8%) and a ventricular septal defect (32.3%). Pulmonary stenosis (20.8%) and aortic stenosis (11.5%) were the most frequent obstructive lesions, while conotruncal and complex malformations each accounted for under 10% of the subgroup. This distribution is qualitatively similar to patterns described in the general CHD literature, where septal and shunting defects collectively constitute the largest category [13,50]. However, direct numerical comparison with registry-based figures—such as the proportion of ventricular and atrial septal defects reported by Schwedler et al. [13] in a national live-birth registry—is not appropriate here, since our percentages were calculated per patient with concurrent lesions (allowing totals above 100%), whereas registry estimates are typically based on a primary or predominant diagnosis per case.

Applying a hierarchical assignment rule based on the most structurally severe lesion, 25 patients (26.0%) were classified as complex CHD and 71 (74.0%) as simple CHD. Patients carried between one and six concurrent lesions (mean 2.55), with a right-skewed distribution in which 60.4% carried one or two defects. Complex CHD patients carried significantly more concurrent lesions than simple CHD patients (median 4, IQR 3–5 versus median 2, IQR 1–2.5; *p* < 0.001). This is the expected consequence of the classification rule combined with the developmental biology of conotruncal malformations, which arise from disrupted neural crest migration and second heart field signaling, and characteristically co-segregate with additional morphogenetic anomalies [51,52,53,54]. The finding should therefore be understood as internally coherent rather than as an independent discovery.

### 4.3. Severity of Growth Restriction and Genetic Syndrome as Baseline Factors Independently Associated with CHD

Within this specification (*n* = 375; χ^2^(5) = 26.46, *p* < 0.001; Nagelkerke R^2^ = 0.100; EPV = 19.2), both severe fetal growth restriction (aOR = 2.01, 95% CI 1.22–3.31, *p* = 0.006) and presence of a genetic syndrome (aOR = 5.97, 95% CI 2.57–13.89, *p* < 0.001) were independently associated with CHD. Gestational age at birth and maternal education did not reach significance, and non-cardiac congenital malformation showed a non-significant inverse trend.

### 4.4. Genetic Syndromes, Extracardiac Malformations and Structural Complexity

Genetic syndromes and non-cardiac congenital malformations were markedly more frequent among CHD-positive than CHD-negative patients on unadjusted comparison (genetic syndrome and Down syndrome, both *p* < 0.001; non-cardiac malformation, *p* < 0.001). Once gestational age, maternal education and FGR severity were accounted for in the prenatal model, genetic syndrome retained a strong independent association with CHD presence (aOR = 5.97, 95% CI 2.57–13.89, *p* < 0.001; Section 4.3), whereas non-cardiac malformation did not (aOR = 0.74, 95% CI 0.34–1.61, *p* = 0.453). We therefore describe genetic syndrome, but not non-cardiac malformation, as an independent baseline factor for CHD in this cohort. For non-cardiac malformation, the unadjusted contrast is best read as reflecting the well-established clustering of cardiac and extracardiac anomalies within syndromic and multi-malformation aggregates [29,30,31,55], with the adjusted model indicating that this particular clustering does not translate into an independent effect at the sample size available.

The genetic contribution was not confined to the presence of CHD: it extended to the structure of the cardiac lesion itself. Within the CHD subgroup (*n* = 96; 25 complex, 26.0%), a multivariable model with an adequate events-per-variable ratio (EPV = 12.5) reached overall significance (χ^2^(2) = 8.34, *p* = 0.015) and identified genetic syndrome as associated with complex CHD (aOR = 5.156, 95% CI 1.651–16.104, *p* = 0.005), while non-cardiac malformation was not (aOR = 2.292, 95% CI 0.592–8.866, *p* = 0.230). Given the modest discrimination (AUC = 0.659; McFadden R^2^ = 0.076), the wide confidence interval and the small number of complex events, this association is exploratory and hypothesis-generating; alternative model specifications were run only as internal consistency checks and do not constitute independent validation. Interpreted cautiously, the convergence of these two findings—genetic syndrome as an independent correlate of CHD presence, and again of CHD complexity, while non-cardiac malformation predicts neither—is consistent with the literature showing that conotruncal and outflow tract defects are disproportionately associated with pathogenic chromosomal variants and with de novo mutations in second heart field transcription factors [48,51], and with the observation that no perinatal or growth-related variable was associated with complexity. The distinction between simple inter-chamber shunting and complex conotruncal malformation reflects qualitatively different embryological pathways, and these pathway-specific disruptions are more closely tied to chromosomal and monogenic determinants than to placental insufficiency [32,56].

### 4.5. Comorbidity Structure of the CHD-Positive and CHD-Negative Subgroups

Digestive and renal comorbidities were significantly more frequent among CHD-negative than CHD-positive patients (15.6% versus 30.8%, *p* = 0.004; and 13.5% versus 35.1%, *p* < 0.001). Because these conditions are ascertained after birth and often after the cardiac diagnosis, they were excluded from the multivariable model, and the contrast is presented descriptively rather than as an adjusted effect. The pattern is most plausibly explained by the competing morbidity structure of a hospitalized FGR population: children admitted with growth restriction but without a cardiac lesion are disproportionately admitted for renal and gastrointestinal problems attributable to the metabolic and nutritional consequences of intrauterine growth failure [3,9], whereas the CHD-positive subgroup is characterized by structural and syndromic morbidity. Nothing in these data supports the inference that CHD is protective against renal or digestive disease; the association is a feature of how patients enter a tertiary hospital cohort.

Consistent with this reading, comorbidity burden did not track structural complexity. Neurologic (31.0% versus 32.0%), digestive (18.3% versus 8.0%) and renal comorbidity (15.5% versus 8.0%) did not differ significantly between simple and complex CHD after Bonferroni correction (all *p* = 1.000). Extracardiac morbidity in this cohort is thus not driven by the anatomical severity of the cardiac lesion. Subtype-level comparisons of genetic syndrome prevalence and comorbidity, including the elevated Down syndrome prevalence observed among patients with complete atrioventricular canal, did not survive correction within the full family of comparisons performed, and are reported as uncorrected exploratory observations rather than as findings.

### 4.6. Hospitalization Outcomes

Diagnosis was established early and almost uniformly across the CHD subgroup: median age at CHD diagnosis was 1.0 month (IQR 0–2), and 95.8% of patients were diagnosed before six months of age. Neither age at diagnosis (*p* = 0.874), rate of early diagnosis (97.2% versus 92.0%, *p* = 0.277) nor length of hospitalization (median 4.75 versus 4.00 days, *p* = 0.119) differed significantly between simple and complex CHD, and the global comparison of length of stay across all subtypes was likewise non-significant (Kruskal–Wallis H = 17.44, *p* = 0.358). We report these as null results and do not interpret the numerical direction of the non-significant differences.

The absence of complexity-related differences is best explained by the structure of the cohort rather than by an absence of clinical impact. Diagnostic timing is subject to a strong ceiling effect, since nearly all patients were diagnosed in the first months of life during an admission undertaken for varied clinical reasons, with echocardiography performed whenever CHD was clinically or antenatally suspected. Length of stay refers to a single index hospitalization and does not capture cumulative healthcare utilization. In cohorts with longer follow-up, complexity-driven differences typically emerge in cumulative inpatient days, surgical interventions and cardiac re-interventions across the first decade of life [57,58,59]—outcomes that the present cross-sectional design cannot address.

Subtype-level differences in length of stay and diagnostic timing were examined but are reported as uncorrected single comparisons that did not survive correction within the defined family of tests; they are not carried forward as findings.

### 4.7. Social and Environmental Factors

Residential milieu (rural vs. urban) showed no significant association with CHD prevalence, complexity, or hospitalization outcomes on bivariate testing, and maternal education level showed no significant independent association with these outcomes in the adjusted models. These findings contrast with reports from lower-income settings where socioeconomic gradients in CHD diagnosis and management access are well documented [25,60], and may reflect the relatively homogeneous socioeconomic composition of our cohort (75.5% rural; predominantly low-to-middle maternal education).

### 4.8. Exploratory Subtype Association Patterns

Hierarchical clustering of subtype co-occurrence produced four empirical aggregates broadly corresponding to endocardial cushion, conotruncal, left-ventricular outflow tract and shunting groupings, and correspondence analysis separated complex and rare lesions from high-prevalence shunting defects along the first dimension. These aggregations are descriptive summaries of co-occurrence in binary data. Intra-cluster Jaccard distances were high (0.811–0.857), indicating limited actual co-occurrence within clusters, and the number of clusters was chosen for interpretability rather than by a stability criterion.

We therefore describe these as empirical subtype association patterns and not as etiologically coherent clusters or clinical aggregates; no stability assessment or external validation was performed. A complementary k-means approximation (k = 3) identified three partially overlapping clinical aggregates (PFO-dominant, ASD + PDA, and multi-shunt profiles) differing descriptively in genetic syndrome burden and length of stay, but with the same modest silhouette score (0.207); both the hierarchical and k-means groupings require confirmation by probabilistic latent class analysis in an independent sample before any biological interpretation is warranted. The one robust structural observation is descriptive and requires no clustering to state: ASD, PDA, PFO and VSD form the densest core of co-occurring lesions in this cohort.

### 4.9. Strengths and Limitations

The strengths of this study lie in the size of the cohort for a single center, the narrow and explicitly stated eligibility criteria, the requirement of echocardiographic confirmation for every cardiac diagnosis, and a conservative case definition of structural congenital heart disease. Eligibility was restricted to children aged 0–18 years with a documented clinical history of fetal growth restriction, born from a singleton pregnancy at 37 to 42 completed weeks. Multiple gestations were excluded by design, and with them the hemodynamic confounding of twin-to-twin transfusion syndrome, as was prematurity, whose effects on growth and cardiovascular physiology would otherwise be inseparable from those of growth restriction. Eight patients in whom an isolated patent foramen ovale was the sole echocardiographic finding were excluded, so that a common physiological variant does not inflate the reported proportion of CHD.

Several limitations qualify the interpretation. First, the study is postnatal and birth-weight-stratified; without first-trimester biometry or systematic fetal echocardiography, the timing of lesion development relative to the onset of placental insufficiency cannot be established. Second, the criteria that strengthen internal validity restrict external validity: the findings apply to term-born singletons and cannot be extended to preterm infants, multiple gestations, or growth-restricted fetuses who did not survive to term. Third, ascertainment was hospital-based within a single tertiary center, with echocardiography performed when CHD was clinically or antenatally suspected; the resulting proportion is not a birth prevalence. Fourth, our case definition limits comparability, since retaining patent foramen ovale only in association with another structural lesion reduced PFO cases from 51 to 43, and series using broader definitions will report higher proportions. Fifth, standardized longitudinal follow-up was unavailable, so for lesions with the potential for spontaneous resolution, particularly patent ductus arteriosus, we could establish neither whether closure occurred nor the age at closure; these were classified on the record available at assessment, without assuming persistence beyond it. Sixth, gestational age was recorded to the nearest completed week and showed near-zero variance owing to scheduled term caesarean deliveries, which precluded its use in some models. Seventh, candidate variables were identified through bivariate screening rather than a prespecified causal framework, so the models are presented as association models and the reported AUC values are apparent, same-sample estimates. Eighth, the complex CHD subgroup comprised only 25 patients and subtype analyses across 17 categories involved very small denominators; comparisons that did not survive correction are labelled as uncorrected and are not carried forward. Additionally, we were unable to restrict PDA cases to those diagnosed outside the expected neonatal transitional period, as diagnosis timing was recorded only at monthly resolution. Excluding the two patients (2.1%) with isolated PDA left Models 1A and 1B materially unchanged (genetic syndrome aOR 6.11 vs. 5.97, and 4.97 vs. 5.16, respectively), indicating our findings are not driven by the inclusion of isolated PDA. Finally, the retrospective observational design permits no causal inference, and all reported relationships—including that between growth restriction and cardiac malformation—should be read as associations rather than as effects in either direction.

### 4.10. Clinical and Research Implications

Both the severity of growth restriction and the presence of a genetic syndrome were independently associated with CHD, and within the CHD subgroup genetic syndrome was further associated with structural complexity. Given the modest internally validated discrimination of Model 1A (optimism-corrected AUC 0.608) and the fact that this study did not directly compare alternative screening strategies or evaluate their clinical utility, these findings should be interpreted as a rationale for prospectively evaluating a targeted echocardiographic approach—rather than as a recommendation supported by the present data. Such an approach could reasonably prioritize FGR neonates with severe growth restriction or a recognized genetic syndrome, the two characteristics independently associated with CHD in this cohort. Non-cardiac malformation, despite its strong unadjusted association with CHD, was not independently associated with CHD after adjustment and is therefore not proposed here as an independent prioritization criterion. We stop short of recommending any screening protocol: the present data derive from a single selected tertiary-care cohort without a population-based denominator, without external validation, and with modest model discrimination, and prospective multi-center confirmation would be required before any protocol recommendation could be supported.

More broadly, the cardiac phenotype of FGR-born children matters beyond the neonatal period. Fetal growth restriction is associated with cardiac remodeling and altered echocardiographic parameters that persist across postnatal age windows [21,61,62], and our group has previously reported long-term metabolic abnormalities and adolescent cardiometabolic risk in FGR populations [9,10]. Whether structural CHD superimposed on this background modifies long-term trajectory is a question the present cross-sectional design cannot answer, and one that argues for prospective longitudinal follow-up of this cohort.

## 5. Conclusions

In this hospital-based cohort of term-born children with fetal growth restriction, echocardiographically confirmed congenital heart disease was documented in a quarter of patients—a proportion reflecting a selected tertiary-care population, not a birth prevalence. Among characteristics available at or before birth, both the severity of growth restriction and the presence of a genetic syndrome were independently associated with CHD, with genetic syndrome showing the stronger association; non-cardiac malformations, although far more frequent among affected children, did not retain an independent association after adjustment. Within the CHD group, a recognized genetic syndrome was also associated with structurally complex disease, while no perinatal, nutritional or social characteristic was associated with complexity or with hospitalization outcomes. Genetic background therefore relates to both whether a cardiac lesion is present and how structurally complex it is, while growth restriction severity relates specifically to the presence of a defect.

These findings support prospectively evaluating a targeted, rather than universal, echocardiographic strategy in term FGR neonates with severe growth restriction or a genetic syndrome—the two factors independently associated with CHD presence in this cohort. Non-cardiac malformation was strongly associated with CHD in unadjusted analysis but did not retain independence after adjustment, and we therefore do not propose it as a basis for prioritization. Given the modest discrimination of the present model and the retrospective, single-center design, prospective multi-center studies with fetal biometry and serial echocardiography are needed to confirm these associations, evaluate the clinical utility of a targeted strategy, and define the longer-term cardiovascular trajectory of this phenotype.

## Figures and Tables

**Table 1:** Baseline characteristics of FGR patients (*n* = 375), stratified by sex and CHD status.

Variable	Total (*n* = 375)	Male (*n* = 174)	Female (*n* = 201)	*p*	CHD+ (*n* = 96)	CHD− (*n* = 279)	*p*
**Demographics**							
**Sex—Male, *n* (%)**	174 (46.4%)	174 (100%)	—	—	44 (45.8%)	130 (46.6%)	0.897
**Milieu—Rural, *n* (%)**	283 (75.5%)	131 (75.3%)	152 (75.6%)	1.000	78 (81.2%)	205 (73.5%)	0.127
**Maternal age (years), mean ± SD**	29.0 ± 7.42	28.29 ± 6.39	29.59 ± 8.18	0.086	29.48 ± 8.11	28.83 ± 7.18	0.487
**Maternal education, *n* (%)**				0.140			0.043 *
**No studies/Primary school**	49 (13.1%)	30 (17.2%)	19 (9.5%)		16 (16.7%)	33 (11.8%)	
**Gymnasium**	119 (31.7%)	56 (32.2%)	63 (31.3%)		25 (26.0%)	94 (33.7%)	
**High school**	176 (46.9%)	72 (41.4%)	104 (51.7%)		52 (54.2%)	124 (44.4%)	
**University**	31 (8.3%)	16 (9.2%)	15 (7.5%)		3 (3.1%)	28 (10.0%)	
**Perinatal Characteristics**							
**GA at birth (weeks), mean ± SD**	38.0 ± 0.29	38.00 ± 0.00	38.05 ± 0.39	0.048 *	38.00 ± 0.00	38.04 ± 0.34	0.338
**Birth weight (g), mean ± SD**	2510 ± 186	2530 ± 182	2490 ± 187	0.013 *	2472 ± 179	2523 ± 187	0.022 *
**FGR severity, *n* (%)**				0.915			0.008 *
**Severe FGR (<3rd centile)**	130 (34.7%)	61 (35.1%)	69 (34.3%)		44 (45.8%)	86 (30.8%)	
**Moderate FGR (3rd–10th centile)**	245 (65.3%)	113 (64.9%)	132 (65.7%)		52 (54.2%)	193 (69.2%)	
**Mode of delivery, *n* (%)**				1.000			0.265
**Vaginal**	18 (4.8%)	8 (4.6%)	10 (5.0%)		7 (7.3%)	11 (3.9%)	
**Cesarean**	357 (95.2%)	166 (95.4%)	191 (95.0%)		89 (92.7%)	268 (96.1%)	
**Nutrition—Feeding Type (first 6 months)**							
**Feeding type, *n* (%)**				0.771			0.310
**Breastfed**	120 (32.0%)	57 (32.8%)	63 (31.3%)		37 (38.5%)	83 (29.7%)	
**Formula fed**	178 (47.5%)	79 (45.4%)	99 (49.3%)		39 (40.6%)	139 (49.8%)	
**Mixed alimentation**	77 (20.5%)	38 (21.8%)	39 (19.4%)		20 (20.8%)	57 (20.4%)	
**Genetics**							
**Genetic syndrome, *n* (%)**	30 (8.0%)	17 (9.8%)	13 (6.5%)	0.325	17 (17.7%)	13 (4.7%)	<0.001 *
**Down syndrome** **(Trisomy 21)**	18 (4.8%)	9 (5.2%)	9 (4.5%)	0.943	12 (12.5%)	6 (2.2%)	<0.001 *
**Other genetic syndrome**	12 (3.2%)	8 (4.6%)	4 (2.0%)	0.239	5 (5.2%)	7 (2.5%)	0.194
**Other congenital malformation (non-cardiac), *n* (%)**	21 (5.6%)	10 (5.7%)	11 (5.5%)	1.000	12 (12.5%)	9 (3.2%)	<0.001 *
**Comorbidities**							
**Neurologic, *n* (%)**	120 (32.0%)	59 (33.9%)	61 (30.3%)	0.531	30 (31.2%)	90 (32.3%)	0.855
**Digestive, *n* (%)**	101 (26.9%)	44 (25.3%)	57 (28.4%)	0.581	15 (15.6%)	86 (30.8%)	0.004 *
**Renal, *n* (%)**	111 (29.6%)	60 (34.5%)	51 (25.4%)	0.070	13 (13.5%)	98 (35.1%)	<0.001 *
**Hospitalization—96 CHD FGR patients**							
**Age at CHD diagnosis (months), mean ± SD**	1.62 ± 2.32	1.27 ± 1.99	1.92 ± 2.53	0.030 * (Mann–Whitney)			
**Diagnosis of CHD before 6 months, *n* (%)**	92 (95.8%)	43 (97.7%)	49 (94.2%)	0.622 (exact Fisher)			
**Length of hospitalization (days), mean ± SD**	5.40 ± 2.99	5.31 ± 2.30	5.46 ± 3.49	0.800 (Welch t)			

GA = gestational age; FGR = fetal growth restriction; CHD = congenital heart defects; LOS = length of hospitalization; SD = standard deviation. Continuous variables: mean ± SD; compared by Welch’s *t*-test. Categorical variables: *n* (%); compared by chi-square or Fisher’s exact test. * *p* < 0.05. No studies/Primary school: merged category (*n* = 49). For multi-category variables, *p* on header row represents the overall test.

**Table 2:** Empirical subtype association patterns of CHD subtypes by Jaccard distance: four descriptive subtype aggregates (*n* = 96).

Cluster	Aggregate Label	Subtypes	Descriptive Rationale	Intra-Cluster Jaccard Dist.	Complexity
**1**	AV Canal & Atrium defects	CAVC (*n* = 6, 6.3%); CA (*n* = 2, 2.1%)	Endocardial cushion defects	0.857	Simple & Complex
**2**	Outflow Tract & Complex	HPA (*n* = 9, 9.4%); DORV (*n* = 2, 2.1%); DCRV (*n* = 2, 2.1%); TGA (*n* = 2, 2.1%); DEXTROC (*n* = 2, 2.1%)	Conotruncal malformations	0.857	Predominantly complex
**3**	Left-sided Obstructions	AOS (*n* = 11, 11.5%); COA (*n* = 6, 6.3%); BAV (*n* = 5, 5.2%)	Left ventricular outflow tract	0.811	Simple & Complex
**4**	Shunting Lesions	ASD (*n* = 44, 45.8%); PDA (*n* = 44, 45.8%); PFO (*n* = 43, 44.8%); VSD (*n* = 31, 32.3%); PS (*n* = 20, 20.8%); TOF/POF (*n* = 8, 8.3%); ASA (*n* = 8, 8.3%)	Inter-chamber & vessel shunts	0.836	Predominantly simple

Jaccard distance = 1 − (patients with both defects)/(patients with either defect). Lower distance = greater co-occurrence. Average linkage method. k = 4 selected for clinical interpretability. Intra-cluster Jaccard distance represents mean pairwise distance within each cluster (lower = more cohesive).

**Table 3:** Multivariable binomial logistic regression—independent factors associated with CHD presence in the full FGR cohort (Model 1A; *n* = 375).

Variable	aOR	95% CI Lower	95% CI Upper	Z	*p*
***Model fit: χ*** **^2^** ***(*** **5*)* = 26.46*, p* < 0.001*|Nagelkerke R*^2^ = 0.100*|AUC* = 0.629 *(bootstrap-corrected* = 0.608*)|AIC* = 412.2*|N* = 375**					
**Gestational age at birth (weeks)**	0.660	0.288	1.513	−0.981	0.326
**Maternal education (ordinal score)**	0.895	0.638	1.255	−0.644	0.519
**Severe FGR (ref: Moderate FGR)**	2.012	1.222	3.313	2.749	0.006 *
**Genetic syndrome (Present vs. Absent)**	5.969	2.565	13.889	4.146	<0.001 *
**Other congenital malformation—non-cardiac (Present vs. Absent)**	0.744	0.344	1.609	−0.750	0.453

aOR = adjusted odds ratio; CI = 95% confidence interval; Z = Wald z-statistic. Outcome: CHD Present (1) vs. CHD Absent (0). Reference categories: Moderate FGR; genetic syndrome absent; other non-cardiac malformation absent. All VIF ≤ 1.05. Apparent Brier score = 0.175; bootstrap-corrected AUC = 0.608, Brier score = 0.181; bootstrap-corrected calibration slope = 0.87; bootstrap-corrected calibration intercept = −0.13. * *p* < 0.05.

**Table 4:** Sensitivity analysis—logistic regression restricted to severe FGR patients (Model 1A subgroup; *n* = 130). FDR-corrected.

Variable	aOR	95% CI Lower	95% CI Upper	Z	*p* (FDR)
***Subgroup: severe FGR patients (n* = 130*; CHD*+ *n* = 44*, CHD*− *n* = 86*)|Model fit: χ*^2^*(*4*)* = 7.38*, p* = 0.117*|McFadden pseudo R*^2^ = 0.045 *(Nagelkerke R*^2^ = 0.077*)|EPV* = 11.0**					
**Gestational age at birth (weeks)**	1.001	0.366	2.739	0.002	0.998
**Maternal education (ordinal score)**	0.712	0.413	1.228	−1.221	0.444
**Genetic syndrome (Present vs. Absent)**	5.303	1.276	22.045	2.295	**0.087**
**Other congenital malformation—non-cardiac (Present vs. Absent)**	0.926	0.333	2.574	−0.148	0.998

Subgroup: aOR = adjusted odds ratio; CI = 95% confidence interval; Z = Wald z-statistic. Outcome: CHD Present (1) vs. CHD Absent (0). FGR severity is excluded as a factor because all patients are severe by definition. BH-FDR correction applied across 4 pre-natal predictors (q = 0.05). Genetic syndrome reached raw significance (*p* = 0.022) but did not survive FDR correction (*p*FDR = 0.087) and is interpreted as exploratory. Apparent AUC = 0.602; bootstrap-corrected AUC = 0.552; apparent Brier score = 0.211; bootstrap-corrected Brier score = 0.229; bootstrap-corrected calibration slope = 0.65; bootstrap-corrected calibration intercept = −0.24.

**Table 5:** Non-cardiac comorbidities by CHD complexity—simple vs. complex CHD, with Bonferroni correction (*n* = 96).

Comorbidity	Simple CHD *n* = 71 *n* (%)	Complex CHD *n* = 25 *n* (%)	*p*	*p* (Bonferroni)	Test
**Neurologic**	22 (31.0%)	8 (32.0%)	1.000	1.000	Chi-square
**Digestive**	13 (18.3%)	2 (8.0%)	0.349	1.000	Fisher exact
**Renal**	11 (15.5%)	2 (8.0%)	0.530	1.000	Fisher exact

Simple CHD = CHD complexity 0 (*n* = 71); Complex CHD = CHD complexity 1 (*n* = 25). Fisher’s exact test or chi-square applied as appropriate. *p* Bonf = Bonferroni-corrected *p*-value (px); corrected significance threshold alpha = 0.017. None of the three comorbidities differed significantly between groups after Bonferroni correction.

**Table 6:** Multivariable logistic regression—factors associated with complex vs. simple CHD (Model 1B; *n* = 96).

Variable	Aor	95% CI Lower	95% CI Upper	Z	*p*
***Model fit: χ*** **^2^** **(2) = 8.34*, p* = 0.015*|McFadden R*^2^ = 0.076*|AUC* = 0.659 *(bootstrap-corrected* = 0.636*)|n* = 96*|Complex CHD n* = 25 (26%)*|EPV* = 12.5**					
**Genetic syndrome (Present vs. Absent)**	5.156	1.651	16.104	2.823	0.005 *
**Other congenital malformation—non-cardiac (Present vs. Absent)**	2.292	0.592	8.866	1.201	0.230

aOR = adjusted odds ratio; CI = 95% confidence interval; Z = Wald z-statistic. Analysis restricted to CHD-positive patients (*N* = 96; Complex CHD *n* = 25 [26%], Simple CHD *n* = 71 [74.0%]). Outcome: Complex CHD (1) vs. Simple CHD (0). Reference categories: genetic syndrome absent; other malformation absent. All VIF ≤ 1.02. Apparent Brier score = 0.175; bootstrap-corrected Brier score = 0.188; bootstrap-corrected calibration slope = 0.93; bootstrap-corrected calibration intercept = −0.06. * *p* < 0.05.

**Table 7:** Hospitalization outcomes by CHD complexity group (*n* = 96).

Group	*n*	Age at CHD Diagnosis—Median (IQR), Months	Early CHD Diagnosis < 6 mo *n* (%)	LOS—Median (IQR), Days	LOS Mean ± SD	*p* (vs. Simple)
**Overall CHD (*n* = 96)**	96	1.0 (0–2)	92 (95.8%)	4.63 (3.37–6.68)	5.40 ± 2.99	—
**Simple CHD**	71	1.0 (1–2)	69 (97.2%)	4.75 (3.69–7.09)	5.66 ± 3.20	—
**Complex CHD**	25	1.0 (0–2)	23 (92.0%)	4.00 (3.00–5.75)	4.64 ± 2.18	LOS: 0.119; Early: 0.277; Age: 0.874

Mann–Whitney U test for continuous outcomes (non-normal distributions confirmed by Shapiro–Wilk). Fisher’s exact test for early CHD diagnosis. mo = months. LOS = length of hospitalization. All patients were hospitalized (no missing data for diagnosis timing). Comparisons: simple vs. complex CHD. *p*-values reported for complex vs. simple CHD comparison.

**Table 8:** Model 1C—Linear regression on age at CHD diagnosis (log-transformed) within CHD-positive patients (*n* = 96). FDR-corrected.

Variable	β	95% CI Lower	95% CI Upper	Std. β	t	*p* (FDR)
***Model 1C: F(*** **7,88*) =* 1.33*, p =* 0.243*|R*^2^ = 0.09*|Adj. R*^2^ = 0.022*|Outcome: LN(age at* CHD diagnosis +0.5*)|n =* 96*|All variables non-significant after FDR. All findings exploratory.***						
**CHD complexity (Complex vs. Simple)**	0.143	−0.231	0.517	0.179	—	0.450
**FGR severity (Severe vs. Moderate)**	−0.187	−0.494	0.120	−0.234	—	0.230
**Genetic syndrome (Present vs. Absent)**	−0.185	−0.592	0.222	−0.232	—	0.369
**Renal comorbidity (Present vs. Absent)**	0.287	−0.153	0.728	0.360	—	0.199
**Neurologic comorbidity (Present vs. Absent)**	0.047	−0.300	0.394	0.059	—	0.789
**Digestive comorbidity (Present vs. Absent)**	−0.003	−0.427	0.421	−0.004	—	0.989
**Sex—Female vs. Male (exploratory †)**	0.361	0.045	0.676	0.451	—	**0.026 †**

Outcome: LN (age at CHD diagnosis in months + 0.5). All VIF ≤ 1.18. Cook’s distance max = 0.121—no influential outliers. BH-FDR applied across 15 secondary comparisons (q = 0.05). No variable survives FDR correction; all findings exploratory. † Female sex: β = 0.361, *p* = 0.026 unadjusted; does not survive FDR. Italic *p* = exploratory (*p* < 0.05 but not FDR-significant).

**Table 9:** Correspondence analysis summary—CHD subtype-by-comorbidity contingency table (*n* = 96).

	Dimension 1	Dimension 2
**Inertia explained**	45.5%	21.5%
**Total inertia (Dim 1 + Dim 2)**	**67.1%**	
**Positive pole**	Complex/rare lesions (DORV, TGA, COA, HPA)	Renal & LVOT comorbidities
**Negative pole**	Common shunting lesions (ASD, PDA, PFO)	Genetic syndrome & Down syndrome
**Clinical interpretation**	Simple ↔ complex CHD axis	Comorbidity type axis
**Cluster quality (silhouette)**	0.207 (poor-to-fair; expected for binary data)—results are hypothesis-generating only	

Correspondence analysis performed on the CHD subtype × comorbidity/outcome contingency table. Inertia = proportion of total variance explained by each dimension. Positive and negative poles indicate the defect subtypes or comorbidity variables driving each dimension. DORV = double outlet right ventricle; TGA = transposition of great arteries; COA = coarctation of the aorta; HPA = hypoplastic pulmonary artery; LVOT = left ventricular outflow tract. Total inertia of the contingency table = 0.235.

**Table 10:** Empirical subtype aggregate profiles identified by k-means latent class approximation (k = 3) within the CHD subgroup (*n* = 96). Hypothesis-generating; not validated clinical aggregates.

Characteristic	Aggregate 1 PFO-Dominant *n* = 35 (36.5%)	Aggregate 2 ASD + PDA *n* = 31 (32.3%)	Aggregate 3 Multi-Shunt *n* = 30 (31.3%)
**Defect profile**			
**Defining defect pattern**	PFO predominant	ASD + PDA co-occurrence	Multiple concurrent shunts
**Mean defects per patient**	1.45	2.1	3.24
**Complex CHD rate**	~26%	~26%	~26%
**Genetic burden**			
**Genetic syndrome**	31.4% ↑	12.9% ↓	Intermediate
**Down syndrome**	Prevalent	Lowest	Intermediate
**Comorbidities**			
**Neurologic comorbidity**	—	—	23.3% ↓
**Digestive comorbidity**	Similar	Similar	Similar
**Renal comorbidity**	Similar	Similar	Similar
**Hospitalization outcomes**			
**Mean LOS (days)**	5.85 ↑	4.73	Intermediate
**Early CHD diagnosis (<6 months)**	Universal	Universal	Universal
**Perinatal characteristics**			
**Birth weight**	Similar across groups	Similar across groups	Similar across groups
**FGR severity distribution**	Similar across groups	Similar across groups	Similar across groups

K-means approximation applied to binary CHD subtype variables (17 subtypes). ↑ = highest value across aggregates; ↓ = lowest value across aggregates. LOS = length of hospitalization; CHD = congenital heart defect; FGR = fetal growth restriction; PFO = patent foramen ovale; ASD = atrial septal defect; PDA = patent ductus arteriosus. Silhouette score = 0.207 (poor-to-fair, expected for binary data); aggregates are hypothesis-generating and require confirmation by probabilistic latent class analysis.

## Data Availability

Data available on request from the first author due to ethical issues.
